# Food additives in childhood: a review on consumption and health consequences

**DOI:** 10.11606/s1518-8787.2022056004060

**Published:** 2022-04-27

**Authors:** Mariana Vieira dos Santos Kraemer, Ana Carolina Fernandes, Maria Cecília Cury Chaddad, Paula Lazzarin Uggioni, Vanessa Mello Rodrigues, Greyce Luci Bernardo, Rossana Pacheco da Costa Proença

**Affiliations:** I Universidade Federal de Santa Catarina Núcleo de Pesquisa de Nutrição em Produção de Refeições Programa de Pós-Graduação em Nutrição Florianópolis SC Brasil Universidade Federal de Santa Catarina. Núcleo de Pesquisa de Nutrição em Produção de Refeições. Programa de Pós-Graduação em Nutrição. Florianópolis, SC, Brasil; II Pontifícia Universidade Católica Faculdade de Direito São Paulo SP Brasil Pontifícia Universidade Católica. Faculdade de Direito. São Paulo, SP, Brasil; III Movimento Põe no Rótulo São Paulo SP Brasil Movimento Põe no Rótulo. São Paulo, SP, Brasil

**Keywords:** Child Nutrition, Eating, Industrialized Foods, Food Additives, toxicity, Review

## Abstract

**OBJECTIVE:**

To discuss the context of scientific publications on the consumption of food additives by children and the possible health consequences in this age group.

**METHODS:**

A literature review, with a search carried out between April 2020 and April 2021 in the Web of Science, Scopus, PubMed and Google Scholar databases, as well as in websites of Brazilian and foreign official bodies. Official documents and studies published since 2000 were selected. Keywords related to food additives, children, food consumption, and health were used for the search.

**RESULTS:**

Food additives are substances intentionally added to foods for technological purposes. Processed foods are the main sources of additives in food and their consumption occurs since childhood. It is observed, however, that there are limitations inherent to the scientific method regarding the analysis of consumption and toxicity of food additives in humans, causing scarcity of data in the scientific literature. Additionally, existing data suggest that the additives have a higher toxic potential in children, considering that the body weight in this age group is lower than in adults. This context emphasizes the need to observe the precautionary principle, according to which risks of harm must be prevented.

**CONCLUSIONS:**

This is a scenario in which the literature points to a risk to people’s health and, in particular, to children, about whom the duty of protection must be even greater, with absolute priority. Thus, the relevance of an expanded technical-scientific debate regarding the establishment of specific and stricter parameters for children is considered, regarding the consumption and toxicity of additives, as well as the different sources of exposure to these substances.

## INTRODUCTION

Studies indicate, in Brazil and other countries, an increase in the purchase of processed foods for consumption from the first months of life^1,2^, especially those classified as ultra-processed^a,3^. In Brazil, soft drinks, industrialized fruit-based drinks (in long-life or powdered packaging), snacks, sweets, chocolates, sausages, breads and cookies are among the most consumed foods by children^7^. These foods usually contain significant amounts of sugar, fat and sodium^17^, and many of them contain food additives^22^, in addition to their packaging often featuring marketing strategies aimed at children^25^.

These additives are not normally consumed as food or used as a typical food ingredient and are intentionally added for technological purposes^26,27^. Criteria for the ingestion and use of additives in processed foods are established worldwide by the *Codex Alimentarius*, a program of the United Nations Food and Agriculture Organization (FAO) and the World Health Organization (WHO), which develops standards and guidelines related to foods and establishes criteria for the ingestion and use of additives in processed foods, through the assistance of an International Scientific Committee, formed by specialists from different countries, called the Joint FAO/WHO Expert Committee on Food Additives (JECFA)^23^.

The expert committee of FAO/WHO (JECFA) analyzes and discusses data from scientific studies on toxicity and safety of additives and, based on these data, the Committee establishes two values for each food additive, designated by the acronyms NOAEL and ADI. NOAEL, acronym for *No Observed Adverse Effect Level*, is the limit amount in which each substance did not show toxic effects in existing studies in the scientific literature. From the NOAEL value, the acceptable daily intake (ADI) is stipulated, the estimated amount in which a substance can be consumed daily, throughout life, without presenting health risks. This value is calculated by dividing the NOAEL value by a safety/uncertainty coefficient, stipulated at 100, which has the purpose of covering potential uncertainties regarding scientific data^23^. That is, it considers possible differences between animal and human models, as well as between sex and age groups, such as different toxicities for children and adults, for example^28^. Thus, the ADI recommended by the *Codex Alimentarius* is, on average, 100 times lower than the amount found to be safe or of low toxicity in scientific studies.

However, there are limitations in assessing the safety of consumption of additives in humans. This is because most studies are performed in animal models or *in vitro*. Authors emphasize that substances react in different ways according to the cellular characteristics of each organism^29^. Moreover, foods are considered complex mixtures of chemical substances, in which different elements, of different molecular weights and chemical configurations, interact with each other and with the organism that ingests them^29^. The level of exposure and individual sensitivity are determining factors to assess whether substances such as additives have toxic potential^30^.

Since the ADI is established per kilogram of weight, the toxicity of additives may be greater in children. Considering their body weight, children drink more water, eat more food and breathe more air than adults. In the first six months of life, children drink seven times more water per kg of body weight and, aged between one and five years, they eat three to four times more food per kg of body weight than the average adult^31^. Furthermore, as they potentially have more years of future life than adults, children have more time to develop chronic diseases triggered by early exposure to environmental substances^31,32^, such as food additives.

It is observed, however, that there are few experimental studies in the scientific literature that evaluate the toxicity of food additives in humans, both in adults and in children, which leads to the need to observe the precautionary principle, according to which the risks of damage must be prevented. This principle, under Brazilian legislation, is based on article 196 of the Constitution, which imposes on the State the duty to guarantee public policies aimed at reducing the risk of disease, in addition to actions and services for the promotion, protection and recovery of health^33^. Also implicit in article 9 of the Consumer Protection Code is the duty to provide information about products that are potentially harmful or dangerous to the health or safety of consumers^34,35^, since access to information is a condition for the conscious exercise of choice by consumers^36^. The issue is particularly relevant given the duty of the State, the family and society to ensure children and adolescents, with absolute priority, the right to life, health, food, etc. as recommended by article 227 of the Brazilian Constitution^33^.

In view of the above, no review studies were found that seek to discuss the methodological challenges involved in research with humans on the consumption of food additives and health effects in children. Thus, the objective of this article is to discuss the context of scientific publications on the consumption of food additives by children and the possible consequences of this consumption for health in this age group.

## METHODS

**Figure 1:**
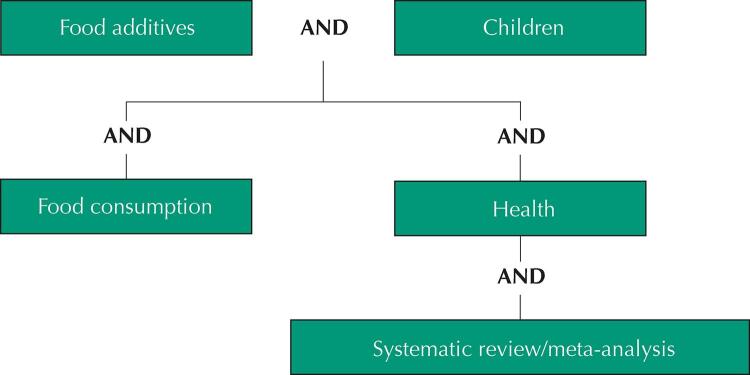
Keyword sets and search strategy.

A narrative review of the literature was carried out, which began with bibliographic searches in the Web of Science, Scopus, PubMed and Google Scholar databases, as well as websites of Brazilian and foreign official bodies, between April 2020 and April 2021. Figure 1 shows the search strategy and the sets of keywords.

Studies published from the year 2000 onwards, as well as official documents on the consumption of food additives by children and their health consequences, were selected and analyzed. In addition, the documents of recommendations and regulations on the intake and use of additives in processed foods were used.

## RESULTS AND DISCUSSION

### Consumption of Food Additives in Childhood

Several methodologies can be used to estimate the consumption of food additives, combining methods of evaluation of food consumption and measurement of food additives content.

Considering food consumption, studies usually use data from population surveys or apply food consumption assessment methods, especially a 24-hour recall, food record and food frequency questionnaire. Based on these data, it is possible to estimate which foods were consumed by individuals and thereby analyze which additives were present in these foods^37^.

As information on the number of additives is not available on food labels, some studies use laboratory analysis for quantification of additives added to foods, with the liquid chromatography technique^38,39^being the most accurate. Other studies infer this amount assuming that the maximum value allowed for each additive was added to the food, as stipulated by the *Codex Alimentarius* (maximum limit) or by the regulatory agencies of the countries. This inference has more limitations, since there is no precision in determining the values and it is not possible to know if the industry used the limit amount allowed, underestimating or overestimating the amount of additive present in the food.

A review study^37^investigated the methodologies used to assess the consumption of additives in the world between 2000 and 2014. Data on the consumption of food additives in all age groups were also analyzed. The studies found focused on four classes of additives: antioxidants, sweeteners, color and preservatives. The quantification of additives in food was performed in two ways: laboratory analysis, usually by liquid chromatography, and content estimation using the maximum permitted limits, with the first methodology being the most frequent in the studies. Food consumption was obtained through population surveys by most studies, the rest used 24-hour recalls and food frequency questionnaires.

The review evaluated 13 studies carried out in nine countries, most of them located in Europe and Asia, which analyzed, primarily in the adult population, the consumption of the sweeteners saccharin, sucralose, aspartame, stevia, acesulfame and cyclamate. Only one study conducted in Canada focused on the consumption of sweeteners by children, which was not higher than the ADI. Six studies analyzed the consumption of sweeteners in different age groups, including children. Of these, a study carried out in India found saccharin consumption values above the ADI by children and adults^37^. No studies found consumption of additives above the ADI of the antioxidants BHA^b^ (buthylated hydroxyanisole), BHT^c^ (butylated hydroxytoluene), and TBHQ^d^ (tertiary butylhydroquinone).

In 18 studies, the consumption of colors, especially tartrazine, sunset yellow, erythrosine and carmoisine, was analyzed. Seven studies carried out in India, one study in Kuwait and one in Thailand found consumption values above the ADI for children. Finally, when analyzing preservatives such as benzoic acid, sorbic acid, sulfites, nitrites and nitrates, the 41 studies conducted in 26 countries found that, on average, the ADI is not exceeded. However, in the highest consumption cases (90th and 95th percentiles), the intake exceeded the ADI, especially by children. Among the conclusions, the authors point out the importance and the need for countries to have mechanisms for monitoring the content of additives used in processed foods^37^.

It is observed in this review study that the consumption of additives by children can exceed the ADI values, especially for colors and preservatives^37^. Remember that these values are stipulated by amount of additive per kilogram of weight. Therefore, it is noteworthy that these parameters can be more harmful to the health of children than adults, in view of the physical and biological factors already discussed. The document General Standard on Food Additives by FAO and WHO^26^, the main recommendation on the subject worldwide, does not provide the value per kilogram of weight to be considered as a basis for calculating the maximum limit. Thus, it is not clear if the amounts considered safe for the addition of additives in food consider the child’s weight and if they are really safe for children to consume.

Official data from the United States show that the use of artificial colors increased, on average, fivefold between 1950 and 2012, from 12 mg to 68 mg per capita per day^40^. In Brazil, authors analyzed in the laboratory the amount of artificial colors present in four types of food: candy, chewing gum, chocolate confectionery and breakfast cereals and the results showed that the colors tartrazine and sunset yellow were the most used. Candies and chocolate confectionery presented amounts of coloring within the permitted range. However, 33% of the chewing gums had amounts of artificial coloring above those allowed by Brazilian Health Regulatory Agency (Anvisa), and one of the brands exceeded fivefold the maximum stipulated limit. On the other hand, all samples of breakfast cereals analyzed showed amounts above those allowed for artificial coloring^41^. It is noteworthy that the analyzed foods are often consumed by the population since childhood. In addition, artificial colors, especially sunset yellow and tartrazine, are the target of toxicological studies that relate them to the development of allergy and hyperactivity symptoms in children^42^.

In addition, the risk from the cumulative consumption of additives, arising from different types of food ingested throughout the day, is questioned. Using the foods analyzed by the study^41^as an example, it is possible to infer that the risk of toxicity by colors seems to be high when a child consumes a breakfast cereal and one or more chewing gums during the day. Considering the other foods consumed each day and the other food additives ingested, the risk of toxicity becomes greater.

In this sense, it is reinforced that children are more vulnerable to the consumption of food additives. As evidenced by the review study^37^, the results on the consumption of food additives differ considerably, depending on the country, the substance analyzed and the methodology used. However, when analyzing the consumption of additives by children, the ADI values are often exceeded.

Box 1 presents the main results of studies found that evaluated the consumption of food additives by children.

**Box 1:** Studies that evaluated the consumption of food additives by children, in chronological order.

Author, year	Country	Consumption assessment	Estimation of additive content	Evaluated additives	Results
Husain et al.^45^ (2007)	Kuwait	24-hour food recall.	Laboratory analysis of 344 food samples.	Artificial colorants: tartrazine, sunset yellow, carmoisine, allura red, orange G, erythrosine, fast green, indigo-carmine, brilliant blue, brilliant black, chocolate brown HT.	Consumption by 3,141 children over 5 years old. In 4 dyes analyzed the consumption was 2 to 8 times above the ADI (tartrazine, sunset yellow, carmoisine and allura red).
Schumann et al.^46^ (2008)	Brazil	Quali-quantitative questionnaire on food frequency of powdered juices, powder for gelatin and soda.	Inference by the maximum limit allowed.	Artificial dyes: sunset yellow, amaranth and tartrazine.	Consumption by 150 children up to 10 years old. Consumption of sunset yellow and amaranth may be exceeding the ADI in 20% and 90% of children, respectively.
Sardi et al.^47^ (2010)	Switzerland	Purchase data provided by a retailer’s card and interviews.	Card from a retail chain, which contains data on the composition of foods.	Sunset yellow dye.	Representative sample of the population with 2,390 individuals of all age groups. In the age group from 1 to 10 years, the average consumption and the maximum values were above the ADI.
Dixit et al.^48^ (2011)	India	Food frequency questionnaire.	Laboratory analysis	Artificial dyes: sunset yellow, erythrosine, tartrazine, carmoisine, ponceau 4R and brilliant blue.	Consumption by 245 individuals aged 4 to 18 years. Considering the average consumption values, erythrosine exceeded the ADI value. Considering the maximum consumption values (95th percentile), in addition to erythrosine, sunset yellow also exceeded the ADI.
Larsson et al.^49^ (2011)	Sweden	Official government data obtained from a 4-day food diary.	Official government data, obtained by laboratory analysis.	Preservatives nitrites and nitrates.	Consumption by 2259 children below the ADI. However, considering the endogenous conversion of nitrate to nitrite, 12% of 4-year-olds may exceed the ADI.
Lok et al.^50^ (2011)	China	Food frequency questionnaire.	Laboratory analysis of 87 foods.	11 artificial colors: tartrazine, quinoline yellow, sunset yellow, amaranth, Chromotrope FB red, ponceau 4R, allura red, erythrosine, indigo-carmine, brilliant blue, lissamine Green B.	Consumption by 142 children aged 8 and 9 years. Mean consumption of sunset yellow dye was higher than the ADI per 9-year-old boy. The consumption of the other dyes did not exceed the ADI.
Polônio e Peres^14^ (2012)	Brazil	Food frequency questionnaire and 24-hour recall.	Inference by the maximum limit allowed.	Artificial dyes: sunset yellow, brilliant blue, amaranth, erythrosine, tartrazine, red 40.	Questionnaire administered to 148 mothers of children between 3 and 5 years old. The consumption of burgundy red and sunset yellow dyes may be exceeding the ADI in 56% and 25% of children, respectively.
Urtiaga et al.^51^ (2013)	Spain	Population survey.	Laboratory analysis of 909 foods.	Sulfite antioxidants.	Consumption of 1,055 individuals aged 4 to 18 years. Consumption was higher than the ADI in 4% of children.
Vin et al.^52^ (2013)	Italy, France, Ireland and the UK	Population surveys.	Inference by the maximum limit allowed and data provided by the industry.	13 additives: benzoates, nitrites, sulfites, butylated hydroxytoluene, polysorbates, sucrose esters, sucroglycerides, polyglycerol esters of fatty acids, stearoyl lactylates, sorbitan esters, phosphates, aspartame and acesulfame.	Consumption by 16,603 individuals of all age groups. Considering consumptions above the 95th percentile, 4 of the 13 additives showed consumption above the ADI in children (sulfites, polysorbates, stearoyl-lactylates and sorbitan esters).
Diouf et al.^53^ (2014)	Germany	Population surveys.	Inference by the maximum limit allowed.	Dyes: carmine, sunset yellow, ponceau 4R, allura red and paprika.	Consumption by 1,234 children aged 6 to 11 years and 1,272 adolescents aged 12 to 17 years. On average, consumption of ponceu 4R exceeded the ADI. Considering the maximum consumptions (above the 95th percentile), the consumption of sunset yellow and ponceau 4R exceeded the ADI.
Mancini et al.^54^ (2015)	France	Population surveys in children under 3 years of age.	Inference by the maximum limit allowed.	Preservatives: benzoates, parabens, nitrites, nitrates; Antioxidants: BHA and BHT; Sweetener: aspartame.	Consumption by 706 children aged 0 to 3 years. Consumption of benzoates, nitrites and BHA exceeded the ADI in, respectively, 25%, 54% and 20% of the population studied.
Suomi et al.^55^ (2016)	Finland	Official government data obtained from a 3-day food diary.	Official government data, obtained by laboratory analysis.	Preservatives: nitrites and nitrates in cured meats and in water.	Consumption by 1,471 children aged 1, 3 and 6 years. Consumption below the ADI for all age groups.
Reddy et al.^56^ (2015)	India	24-hour recall.	Laboratory analysis.	Preservatives: sodium benzoate and potassium sorbate.	Consumption by 960 individuals aged 2 to 19 years. Consumption below the ADI.
Martyn et al.^57^ (2016)	Ireland	Official government data.	Official government data, obtained by laboratory analysis.	Sweeteners: acesulfame K, saccharin, aspartame and sucralose.	Consumption by 500 children aged 1 to 4 years. Consumption below the ADI.
Feitosa et al.^58^ (2017)	Brazil	Official government data.	Inference by the maximum limit allowed.	Sunset yellow dye.	Consumption by a representative sample of the population over 10 years old. Consumption above the ADI for children over 10 years old, considering the prevalence of consumption of the analyzed foods.
Bastaki et al.^59^ (2017)	USA	Official government data.	Data provided by the industry.	Dyes: allura red, tartrazine, brilliant blue, sunset yellow, indigo-carmine, erythrosine, fast green.	Consumption by 16,011 individuals of all age groups. Consumption below the ADI for all age groups.
Choi e Suh^60^ (2017)	Korea	Population survey.	Laboratory analysis of 287 foods.	Nitrite preservative.	Consumption by 8,019 individuals of all age groups. Average consumption below the ADI.
Martyn et al.^61^ (2017)	Brazil, Mexico, Canada, USA	Population surveys of the 4 countries.	Data provided by the industry.	Benzoate preservatives in non-alcoholic beverages.	Consumption by a representative sample of the population of the 4 countries. Consumption may exceed the ADI above the 95th percentile in Canada and Mexico.
Bel et al.^62^ (2018)	Belgium	Official government data.	Inference by the maximum allowable limit and laboratory analysis.	Emulsifiers: sodium and calcium stearoyl-2-lactylate.	Consumption by a representative sample of the population of all age groups. Maximum limit: consumption of 92% of children possibly exceeds the ADI; Laboratory analysis: consumption exceeded the ADI in 1.9% of children.
Garavaglia et al.^63^ (2018)	Argentina	Population survey.	Food labeling and data provided by the industry.	Sweeteners: acesulfame K, saccharin, aspartame, cyclamate and sucralose.	Consumption by 2,664 individuals aged 2 to 18 years. Average consumption below the ADI. Considering maximum intakes, 0.3% of children exceeded the ADI for saccharin and 0.9% for cyclamate.
Long et al.^39^ (2019)	Vietnam	24-hour recall.	Laboratory analysis.	Preservatives: benzoates and sorbates; Sweeteners: cyclamate and saccharin; Dyes: tartrazine and sunset yellow.	Consumption by 10,499 individuals of all age groups. Benzoate consumption was higher than the ADI in 4.6% of children under 5 years of age and 2.6% of children between 6 and 10 years of age.
Martínez et al.^64^ (2020)	Chile	Food frequency questionnaire.	Food labeling and data provided by the industry.	Sweeteners: acesulfame K, stevia, saccharin, aspartame, cyclamate and sucralose.	Consumption by 250 children between 6 and 12 years old. Consumption below the ADI. However, all 250 children evaluated consumed at least one type of sweetener daily.

ADI: acceptable daily intake; BHA: acronym for buthylated hydroxyanisole; BHT: acronym for butylated hydroxytoluene.

We found 22 studies, carried out in 21 countries, that analyzed the consumption of six functional classes of additives by children: colors, preservatives, sweeteners, antioxidants, emulsifiers and stabilizers. It is noteworthy that the colors, especially sunset yellow and tartrazine, were the most studied. In sixteen studies, at least one additive had consumption estimated to be above the safety limits, of the following functional classes: colors^14^, preservatives^39,49,54,61^, antioxidants^51,52,54^, emulsifiers^62^and sweeteners^63^.

In 13 studies, food consumption was analyzed through previously collected population surveys. Official government data, food frequency questionnaire, 24-hour recall and purchase information in supermarket chains were also used. In 10 studies the additives were quantified through laboratory analysis and the rest inferred the amounts by the maximum limit allowed (seven studies) and information provided by the industry (five studies).

Four studies were found in Brazil: one on preservatives (benzoates) and three on colors. Martyn et al.^61^ (2017) used consumption data from the 2008/2009 Family Budget Survey (POF) of the Brazilian Institute of Geography and Statistics (IBGE), with 34,003 individuals over 10 years of age, and did not identify values above the ADI for benzoates, quantified from contact with manufacturers. However, Schumann et al.^46^ (2008), Polônio and Peres^14^ (2012), as well as Feitosa et al.^58^ (2017), found consumption values above the ADI for the sunset yellow, burgundy red, and amaranth colors. These three studies estimated the amount of food additives using the maximum allowable limits. However, while Schumann et al.^46^ (2008) and Polônio and Peres^14^ (2012) administered a food frequency questionnaire to the participating children, Feitosa et al.^58^ (2017) used consumption data from the 2008/2009 POF/IBGE.

As in Brazil, the consumption of colors seems to exceed the ADI in Kuwait^45^, Switzerland^47^, India^48^, China^50^and Germany^53^. In the analysis of preservatives consumption, the results indicate consumption both above and below the ADI. Unlike Brazil^61^, in Vietnam^39^, Canada^61^, Mexico^61^and France^54^ the consumption of preservatives exceeded the ADI. Regarding the other classes of additives studied, there is evidence of possible excessive consumption of antioxidants^51,54^and emulsifiers^62^by children.

Based on the data available in the scientific literature, summarized in Box 1, a possible high consumption of additives in childhood is highlighted, according to the ADI values stipulated by the *Codex Alimentarius*, especially for colors. Additionally, it is noteworthy that no article was found that analyzed the cumulative consumption by children of different additives over time. This gap in the scientific literature is relevant, given that, throughout each day, individuals consume multiple servings of different types of food that can potentially be sources of different additives.

Corroborating this statement, one of the objectives of the NutriNet-Santé cohort study, carried out in France with 106,000 adults, was to describe the exposure profiles to different additives by the population (one additive or mixtures of different types of additives). Five groups were found, composed of different foods. The first group comprises additives found in cookies and cakes (lecithins, mono- and diglycerides of fatty acids, carbonates, diphosphates, glycerol and sorbitol), consumed mostly by non-smokers with graduate degrees, with the highest energy and lipid consumption averages. The second group corresponds to additives found in broths, butter, breads and meal replacements (modified starches, monosodium glutamate, fatty acid esters and BHA), consumed by physically active, older non-smokers. Then the third group of additives found in dairy desserts, breakfast cereals and baked goods (carrageenan, lactic acid, calcium propionate and phosphates), consumed by people with the highest carbohydrate intakes. The fourth group concerns the additives found in sauces and processed meats (sodium nitrite, sodium erythorbate, phosphates and cochineal), often consumed by men with the lowest levels of education. Finally, the fifth group refers to additives found in sugary and artificially sweetened beverages (mixture of sweeteners – acesulfame K, aspartame, sucralose, steviol glycosides – colors, pectins, carotenes, sodium citrate, benzoates, phosphates, nitrates), consumed by younger individuals, with higher body mass indexes, lower levels of physical activity and more likely to be smokers^65^.

On the other hand, this study highlights a sixth group, related to the lower consumption of additives, found in whole foods, legumes, breakfast cereals without added sugar, vegetable juices, oilseeds, vegetable oils and cheeses. This food group was mostly consumed by women, with the lowest consumption of energy and ultra-processed food and the highest consumption of organic foods and alcoholic beverages. As conclusions, the authors highlight that the health impact and potential effects of the consumption of different types of additives should be explored in epidemiological and experimental studies. Following the precautionary principle, several public health authorities around the world have recently started to recommend the consumption of foods without or with as little additives as possible^65^.

It was observed that the studies presented in Box 1 analyze food consumption and quantify food additives using different methodologies. The main methods of assessing food consumption were a 24-hour recall, food purchase data and a food frequency questionnaire. The amount of additives in food was identified by laboratory analysis, data provided by the industry or inferred by means of an estimate by maximum limit. This scenario of little standardization in the method of data collection and analysis may indicate a methodological limitation in studies on the subject, insofar as the results of the studies cannot be compared with each other, weakening the existing scientific evidence on the consumption of additives.

It should be noted that there are additives that do not have maximum limit values determined due to the absence of an ADI established by JECFA and, therefore, their use is recommended by the *Codex Alimentarius* and/or authorized by the regulatory agencies of the countries on a *quantum satis* basis. This term means that the manufacturer is allowed to add the amount of additive that he deems necessary and sufficient to achieve the intended technological function, with no maximum value determined for addition at the time of manufacture (maximum limit). As an example, in Brazil, Anvisa authorizes in bakery products and cakes, among other additives, the use of the emulsifier soy lecithin and all flavorings in the *quantum satis* amount^66^. With this, it is inferred that it is only possible to analyze the effective consumption of these additives through laboratory analysis or contact with the industry, limiting the performance of studies on such substances.

However, although the *quantum satis* limit is authorized in Brazil and validated by the *Codex Alimentarius*, the subjectivity of the definition of this amount of additive to be added to foods is conjectured, as well as the potential risks, with the understanding that the manufacturers are authorized to add the amount of additive they deem necessary, without necessarily considering the safety of consumption of the substance. Furthermore, it should be noted that the consumer does not have any mechanism for accessing information, neither regarding the permitted amount of use nor the amount actually added to the food. This is because the current legislation indicates that food additives must be declared after the ingredients (and not in descending order of proportion, as is the case with ingredients).

In addition to the lack of consumer information on labels, this context can therefore lead to inaccuracies in the quantification of additive consumption. Consequently, it can bring limitations not only for the analysis of additive consumption, but also for the assessment of toxicity and health effects in humans.

### Food Additives and Consequences for Children’s Health

The consumption of ultra-processed foods may be directly related to the development of obesity, diabetes, cancer and other chronic noncommunicable diseases^67,68^. However, it is still uncertain which variables present in ultra-processed foods most contribute to these results, citing the need to better analyze food additives, among other components^69^.

Most studies to identify the toxicity of food additives are carried out with rodents in the laboratory^70^, which must follow design and execution protocols so that their results are validated by the *Codex Alimentarius* and by regulatory agencies around the world when establishing the ADI. The main protocol used comes from the Organization for Economic Cooperation and Development (OECD) guidelines for chemical testing, which comprise around 150 internationally agreed methods to identify and characterize the potential hazards of chemicals^85^. Although there are strict methodological protocols for experimental studies, it is questionable, in the light of scientific methodology and the precautionary principle, whether it is appropriate to extrapolate results found in cells or animal models to humans, in order to establish limits for human consumption for potentially toxic substances, such as additives.

In Technical Report No. 70 of 2016^86^, which aimed to clarify questions about the declaration and content claims for food additives in food labeling, Anvisa states that:

Although food additives are subjected to a safety and technological efficacy assessment prior to authorization of use, the globally accepted approach used in safety assessment has several limitations, such as: the difficulty in transposing toxicological data obtained in studies with animals to humans and the difficulty of predicting inter-individual variability. Furthermore, new studies have suggested that these substances may cause adverse reactions not identified in the safety assessment, including allergic reactions, food intolerances and hyperactivity (...)

However, it is these toxicological evaluation studies, carried out mainly in animal models, that support the *Codex Alimentarius* in the ADI and maximum limits recommendations in foods. This aspect is considered a methodological limitation in studies on health effects in humans, since, even properly performed, the effects (or lack of them) found in animals will not necessarily occur in humans or at the same intensity.

Dybing et al.^29^ (2002) emphasize that, although methodologically there are formulas to extrapolate the results to human beings, it is known that substances react in different ways according to the cellular characteristics of each organism. In addition, authors question the use of the NOAEL value as a reference to support ADI recommendations for additives. The sample size of the studies is considered a sensitive point, as they vary between them and because, often, there are small samples to consider that a substance does not have toxic effects^87^. Furthermore, they consider that the determination of the NOAEL value does not consider the progression of the toxic effect in relation to the duration and/or dose of the additive^88^.

As an example regarding the controversies and methodological challenges to attest to the toxicity of food additives, the recent discussion regarding the use of titanium dioxide in foods stands out. The use of this dye is attested by JECFA in the *quantum satis* limit since 1969, the year of the last toxicological evaluation carried out by the committee. In this analysis, the studies did not demonstrate toxic effects of titanium dioxide in animal models. However, in March 2021, the European Food Safety Authority (EFSA) published a new toxicity assessment and concluded that the additive should no longer be considered safe for human consumption, in any quantity^89^. This debate began with a position taken by the French regulatory agency (*l’Agence Nationale de Securité Sanitaire de l’Alimentation* – ANSES) which, after an analysis carried out by experts, published a decree suspending the marketing of foods containing titanium dioxide, as of January 1, 2021, for not considering this additive safe for human consumption^90^. Thus, the use of titanium dioxide has been (re)discussed in several countries around the world, also being included in Anvisa’s Food Regulatory Agenda 2021/2023^91^.

In this context, safety and toxicity assessments are generally performed with only one additive, underestimating the effects of associating two or more substances, which may interact when ingested^92^. Thus, the cumulative and concomitant consumption of different types of additives is another latent aspect regarding toxicity. The interaction of different additives together, both with each other and with the human organism, is little studied. Therefore, the relevance of this issue in the establishment of the ADI of food additives is uncertain. In a study carried out with 50 Wistar rats, for example, the effect of the concomitant consumption of different types of additives (colors, preservatives and sweeteners) on blood markers and on liver, kidney and brain tissues was evaluated. The additives present in foods consumed by children and that were the subject of controversies regarding safety of consumption were chosen. As a result, the authors point out that, although the NOAEL value established for each additive separately appears to be safe, when different types of additives are consumed together, this safety can be compromised. Consumption of different types of preservatives and, concomitantly, of preservatives, colors and sweeteners demonstrated potential risks of damage to the DNA of brain, kidney and liver cells. In addition, as the number of administered additives increased, there was a reduction in the levels of hemoglobin, albumin and total serum protein, as well as an increase in urea, creatinine, bilirubin and liver enzyme activity. These changes can trigger various metabolic damages, as well as diseases resulting from DNA damage and imbalances in biochemical parameters^83^.

**Figure 2:**
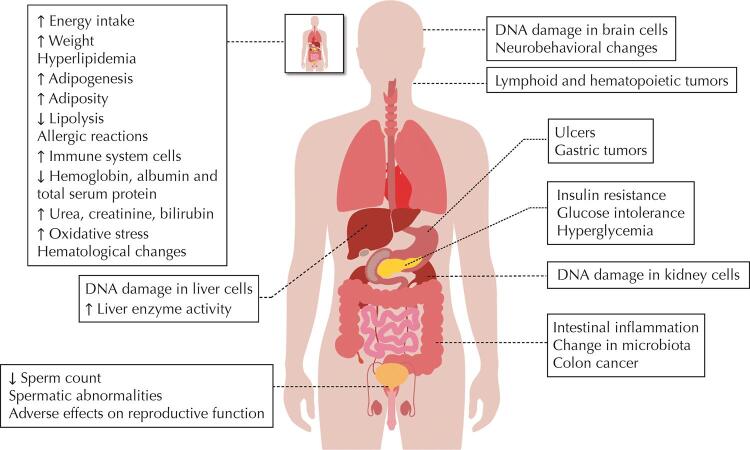
Diseases identified in animal models resulting from the consumption of food additives.

Figure 2 presents the results associating toxicity of food additives in animal models^70^.

A systematic review study^93^on the potential risks of benzoate and sorbate preservatives indicated that, in isolation, these substances do not seem to have toxic effects in mammals. However, in contact with other additives in the gastric environment, such as nitrites and ascorbic acid, they can form substances with carcinogenic potential. Furthermore, results in animal models indicate potential teratogenic effects and liver damage; deleterious effects on neuronal development and growth retardation, hematological abnormality and organ damage. The authors of the review also discuss that in studies carried out *in vitro*, there are results indicating increased oxidative stress, damage to genetic material, inhibition of leptin release in adipocytes and mitochondrial damage^93^.

In humans, most of the review studies found that evaluated the possible health consequences of the consumption of additives are inconclusive. Possibly, inconclusive results occur due to the impossibility of comparison determined by the different methodologies used. In this sense, studies that evaluated the consumption of artificial sweeteners and metabolic effects^94,95^, as well as nitrites and nitrates and cancer^96,97^stand out. These studies point to the need for further investigations in humans to draw conclusive results.

A scoping review aimed at mapping possible health outcomes associated with frequent consumption of artificial sweeteners found 372 articles that investigated various health changes, such as: cancer, diabetes, changes in appetite, caries, weight gain, obesity, headache, depression, behavioral and cognitive effects, neurological effects, risk of preterm birth, cardiovascular effects and risk of chronic kidney disease. However, the authors consider the results to be inconclusive and point to the need for more research, especially longitudinal studies with rigorous and detailed methodological procedures, as well as well-executed systematic reviews, allowing quantitative summary and validity analysis of existing data^98^.

Other review studies point out that, although there is no conclusion that confirms the relationship between additives consumption and health outcomes, there is also no evidence to reject it^99^. It is known that the methodology of experimental and observational studies must be carefully analyzed to verify if there was methodological rigor that scientifically validates the results. However, data obtained from primary studies should be considered, especially by public health bodies and regulatory agencies. Additionally, in view of the lack of consensus in the scientific literature regarding damage to health, especially for potentially toxic substances, such as food additives, the precautionary principle should be considered, which provides, when there is no scientific proof of safety, the adoption of measures against potential risks whenever there is a danger of serious or irreversible damage^102^.

Although many studies do not find conclusive results, there are reviews in the scientific literature that, when evaluating primary experimental and/or observational studies, point to the relationship between consumption of additives by humans and potential damage to health. Given the scarcity of review studies with this objective, Box 2 summarizes the results found, both in children and adults.

**Box 2:** Review studies, with conclusive results, that analyzed the effects of additives consumption on the health of adults and children.

Authors/year	Methodology	Additives	Health effects
**Adults**			
Vally et al.^103^ (2009)	Literature review	Sulfites	Breathing problems
Song et al.^104^ (2015)	Meta-analysis that included 22 articles consisting of 49 studies - 19 studies for nitrates, 19 studies for nitrites and 11 studies for *N*-nitrosodimethylamine.	Nitrates, nitrites and nitrosamines .	Development of gastric cancer .
Romo-Romo et al.^105^ (2016)	Systematic review that included 14 observational and 28 experimental studies. Meta-analysis with two experimental studies.	Sweeteners	Metabolic dysregulation.
Paula Neto et al.^92^ (2017)	Literature review.	Citrate, artificial sweeteners, carrageenan, emulsifiers.	Change in microbiota and metabolic dysregulation.
Azad et al.^106^ (2017)	Systematic review with meta-analysis that included 7 experimental studies and 30 cohort studies.	Sweeteners	Weight gain and cardiometabolic effect.
Crowe et al.^107^ (2019)	Literature review	Sodium nitrite	Development of colorectal cancer.
**Children**			
Schab e Trinh^42^ (2004)	Systematic review with meta-analysis that included 15 experimental studies with children.	Artificial dyes	ADHD
Polônio e Peres^43^ (2009)	Systematic review that included 13 cross-sectional and experimental studies with children.	Artificial dyes	Rhinitis, urticaria and angioedema.
Kanarek^44^ (2011)	Literature review of experimental studies with children.	Artificial dyes	ADHD

ADHD: attention deficit hyperactivity disorder.

Nine studies were found, of which six analyzed the health effects in adults^92,103^and three in children^42^. The results seem to point to the potential for the development of attention deficit hyperactivity disorder (ADHD), alterations in the intestinal microbiota, metabolic dysregulation, weight gain, cardiometabolic effects, development of cancer in the gastrointestinal tract, respiratory problems, rhinitis, urticaria, and angioedema. In addition, studies have looked at the health effects of different types of additives, such as: sulfites, nitrites, nitrates, nitrosamines, sweeteners, carrageenan, citrate, and emulsifiers.

In children, the review studies identified analyzed the health effects caused by only one functional class of additives, the colors. Three studies were found, associating its consumption with short- and long-term effects on the development of allergic reactions, such as rhinitis, urticaria and angioedema, as well as behavioral disorders, such as ADHD. Schab and Trinh^42^ (2004) point out that artificial colors promote hyperactivity in children, considering symptoms measured by behavioral assessment scales. Kanarek^44^ (2011), when analyzing the same variables, highlights that, although the consumption of colors seems to be associated with the worsening of symptoms of hyperactivity and/or attention deficit, their complete withdrawal from food may not be enough for the treatment of ADHD symptoms, considering the multifactorial nature of the causes.

Polônio and Peres^43^ (2009), emphasize that the number of studies was greater, and the results were more consistent regarding the clinical manifestations of non-specific hypersensitivity, such as rhinitis, urticaria and angioedema, related to the consumption of artificial colors. However, they also point out that, although with divergent results, studies have found a relationship between the consumption of additives and the development of cancers, especially when consumption was higher than the ADI.

Although no review study was found with conclusive results about the effects of sweeteners on children’s health, Shum and Georgia^108^ (2021), in their review, emphasize that the consumption of this additive seems to be frequent in this age group and, sometimes, higher than the recommended limits. Thus, they point to the need for studies regarding the potential effects on children’s health, especially regarding the possible risk of developing type 2 diabetes and cardiometabolic diseases resulting from the consumption of sweeteners; they also point out the importance of investigating how intrauterine exposure to sweeteners can influence metabolic outcomes during life.

In this sense, a systematic review with meta-analysis analyzed the effects of maternal consumption of sweeteners on outcomes during birth, specifically birth weight, preterm delivery and gestational age. The authors emphasize that the evidence is of low quality; however, it suggests that the daily consumption of sweeteners during pregnancy is associated with an increased risk of preterm birth, decreasing gestational age and increasing birth weight^109^.

There are few experimental studies relating the effects of additives consumption to the health of children, but there are hypotheses under study. The most cited research and that has produced the most robust results to date was carried out in England and published in the *Lancet* journal in 2007, by McCann et al.^110^ (2007). It is a randomized, placebo-controlled, double-blind clinical trial. The authors evaluated the effect of consuming two beverages containing different concentrations of food additives, compared to placebo, on behavioral outcomes of hyperactivity in children aged three to four years and eight to nine years. Both drinks contained artificial colors (sunset yellow, carmoisine, tartrazine and ponceau 4R), in higher concentration in the second drink, and sodium benzoate preservative in the same concentration in both drinks. As a result, consumption of both beverages, in both age groups, increased the mean level of hyperactivity in relation to placebo, correlating the consumption of artificial colors and sodium benzoate with the behavioral outcome in children^110^. It is noteworthy that there were criticisms of the study methodology, especially the dose of additives used^111^. However, McCann et al.^110^ (2007) indicate in the method of the article that the doses used in drinks for children aged three to four years correspond to the consumption of two packages of 56g candies. The amounts of additives present in one of the eight- and nine-year-olds’ drinks correspond to four packets of candy. Initially, the question is, which child within the age groups surveyed would habitually consume this amount of candies?

Randomized clinical trials are known to provide high levels of scientific evidence if properly performed. In addition, this study design usually has space for publication in journals with a high impact factor, as in the aforementioned study^110^. However, the discussion about ethical aspects involved in the design and execution of this type of study is considered relevant, when the main outcome is the effect of the ingestion of substances potentially harmful to the body.

This situation is even more latent when the target of the studies is children. First, authorization from those responsible for the participation of children in any type of study is required. It is questioned whether all the risks involved in the administration of potentially toxic substances, such as additives, are fully explained to those responsible for authorizing the participation of a child in a study with this design, in which there are risks involved and, certainly, the child you will not have any health and well-being benefits from participating. In addition, consideration is given to the harmful and permanent effects that can be generated to participants in experimental studies that analyze the toxicity of ingested substances. It is considered that the responsibility of the researchers regarding the possible consequences for the study participants and the ethical aspects involved in experimental designs that assess toxicity may be limitations for the development of research in this area.

In a report by the American Academy of Pediatrics, Trasande et al.^40^ (2018) discuss the results of studies on the consequences of consuming nitrite and nitrate preservatives on children’s health. The authors argue that some evidence points to the action of these preservatives as endocrine disruptors, altering thyroid metabolism and the interaction with other substances in the body (amines and amides) forming carcinogenic compounds, mainly in the brain and gastrointestinal tract. This situation can be potentiated in the organism of infants and young children, due to the immaturity of the organism. There is even evidence that highlights the relationship between maternal consumption of nitrites and nitrates with the development of brain cancer in babies .

Occurrences of allergic reactions in children due to the consumption of additives have already been scientifically published in clinical reports, mainly associated with preservatives of the benzoate class^112,113^, as well as with colors^114^. The consumption of colors, specifically, can activate the inflammatory cascade, resulting in the induction of intestinal permeability to large antigenic molecules. In addition to allergic reactions, intestinal permeability can lead to autoimmune diseases and neurobehavioral disorders^115^. A clinical report on the subject points out that there are no data on the prevalence of allergy to food additives in children, which makes the diagnosis difficult. However, this relationship should be clinically investigated whenever the patient is allergic to multiple foods and medications^116^.

There are also observational studies (population and cohort) that found possible correlations between: consumption of artificial sweeteners and early menarche^117^; consumption of artificial sweeteners by pregnant women and excessive weight gain in babies up to one year of age^118^; and risk of overweight in seven-year-old children^119^; as well as consumption of monosodium glutamate, aspartame and nitrites as triggers for headaches in children^120^.

Due to the already discussed ethical issue of the unsuitability of carrying out experimental studies offering potentially toxic additives to human beings, longitudinal observational studies are the most important sources of evidence gathering on the subject. However, the impossibility of inferring a causal relationship in this study design is highlighted, as well as the difficulty of separating the health effects arising from the additives from the other components of the foods that contain them.

As previously stated, most of the review studies found on additives consumption and human health address the effects of the consumption of sweeteners and preservatives in adults, while in children only the effects of artificial colors were analyzed. The most related health outcomes in children were behavioral and immunological disorders, although in adults, studies point to other possible consequences, such as the development of cancers in the gastrointestinal tract, metabolic dysregulation, weight gain and cardiometabolic effect. However, considering that there are hundreds of additives allowed for use in the world, a minimal portion of these substances are studied and tested in humans, especially in children. In addition, no studies were found that evaluated the health impact due to the regular and cumulative intake of food additives in humans.

When it comes to children, the context of consumption recommendations and the assessment of additive toxicity is even more complex, as an important aspect, the initial stage of life, is not considered when establishing recommendations. The ADI, maximum consumption parameter for, ideally, no toxic effect, is established by milligrams of additive per kilogram of weight, but it is not clear which kilogram of weight value is used as a reference to establish this parameter. Thus, it is questioned whether the mg/kg weight ratio is applied by processed food manufacturers, considering an average child weight or, as a consequence, the greater toxicity of food additives in children. When dealing with additives without an established ADI, this situation becomes even more worrying. In these cases, their addition to foods must follow good manufacturing practices, that is, additives can be added in a *quantum satis* amount, which is the smallest amount possible to achieve the desired technological effect, without altering the identity and genuineness of the food, according to identity and quality standards determined by specific regulations^24,66^. In such cases, it is not possible to identify what amount of additive is added to the food and whether this amount can be toxic for children, and it is unknown what the effects are of the combination of these additives with each other and for which the legislation provides a maximum limit of use.

## CONCLUSIONS

It is a scenario in which the literature points to a risk to the health of people and, in particular, children, whose duty of protection must be even greater, with absolute priority. However, the establishment of an additive consumption limit, or ADI, is carried out considering effects identified in toxicological studies carried out, mostly, in animal models. When applied to children, the context of consumption limits and the assessment of toxicity of additives are more complex, since an important aspect, the initial stage of life, is not considered when establishing safety limits. It is known that the toxicity of food additives is greater in children, because the amount ingested per kilogram of weight is greater. Furthermore, organs and systems are still being formed at this stage of life, exposing children to potentially greater health risks that can arise from additives consumption. In addition, the level of exposure throughout life may be higher in children today, since they started to consume processed foods and food additives in the first years of life^31,32^.

In this sense, the existing limitation in the scientific method for carrying out toxicity studies of potentially toxic substances in humans, especially in children, is considered evident. Additionally, it is noteworthy that there are methodological limitations for the evaluation of children’s additives consumption, firstly, because the methods for evaluating food consumption are diverse and not always comparable, in addition to the fact that the quantification of additives in food is performed in different ways, with laboratory analysis being considered the gold standard. However, many studies estimate the amount of additives in foods, through the maximum limit allowed for each substance, causing methodological differences that make it difficult to compare the results across studies, as well as to analyze methodological quality. Thus, it is understood that this context contributes to the fragility of the existing evidence, as well as to the scarcity of discussions on the subject.

Based on the precautionary principle, it is up to the State to promote measures aimed at protecting the health of the population (including risk, under the terms of article 196 of the Constitution and article 9 of Consumer Protection Code), which results in the duty to promote public debate on the subject and public policies that allow access to information on the amount of additive used in food, so that people can make informed and conscious choices.

It is observed that studies on additives consumption, as well as those that evaluated health consequences, focus their analyses on three functional classes: colors, sweeteners and preservatives. However, the representativeness of the additives studied in relation to the total number of additives allowed for use is questioned. In Brazil, there are 23 regulated functional classes and hundreds of Anvisa norms that establish which additives and in what quantity can be used in foods^e^. This context makes it impossible to accurately analyze how many additives are allowed for use in the country, so that it becomes possible to verify the scenario of scientific discussions on the subject. Additionally, the analysis of the notification of additives on processed food labels is scarce in Brazil and in the world. Through these data, it would be possible to assess which additives are used most frequently in processed foods and, thus, relate data on frequency of use, consumption and health consequences.

Finally, additives, such as colors and sweeteners, are present not only in foods, but also in medicines and oral hygiene products, and can be ingested through different sources. Thus, the relevance of an expanded technical-scientific debate regarding the establishment of stricter parameters of consumption and toxicity of specific additives for children is appreciated, considering the different sources of exposure to these substances.

## Footnotes

a Part of the so-called NOVA classification of foods. Ultra-processed foods are usually made from multiple ingredients. These foods may contain ingredients extracted from other foods (casein, gluten, etc.) or derived from other foods (maltodextrin, invert sugar, hydrogenated oils, etc.). In addition, food additives such as dyes, sweeteners, stabilizers, flavor enhancers, among others, are used intensively.

b Acronym for *buthylated hydroxyanisole*

c Acronym for *butylated hydroxytoluene*

d Acronym for *tertiary butylhydroquinone*

e Anvisa is in the process of compiling the rules that regulate the use of additives in processed foods in Brazil.
