# Supplementary material for: Food additives in childhood: a review on consumption and health consequences
Source: Rev Saude Publica. 2022 Apr 27;56:32. doi: 10.11606/s1518-8787.2022056004060 (PMC9060765; doi:10.11606/s1518-8787.2022056004060)
Supplement: Translation

# Translation: Aditivos alimentares na infância: uma revisão sobre consumo e consequências à saúde

**Authors:** Mariana Vieira dos Santos Kraemer, Ana Carolina Fernandes, Maria Cecília Cury Chaddad, Paula Lazzarin Uggioni, Vanessa Mello Rodrigues, Greyce Luci Bernardo, Rossana Pacheco da Costa Proença

**Affiliations:** I Brasil Universidade Federal de Santa Catarina. Núcleo de Pesquisa de Nutrição em Produção de Refeições. Programa de Pós-Graduação em Nutrição. Florianópolis, SC, Brasil; II Brasil Pontifícia Universidade Católica. Faculdade de Direito. São Paulo, SP, Brasil; III Brasil Movimento Põe no Rótulo. São Paulo, SP, Brasil

**Keywords:** Nutrição da Criança, Ingestão de Alimentos, Alimentos Industrializados, Aditivos Alimentares, toxicidade, Revisão

## Abstract

**OBJETIVO:**

Discutir o contexto das publicações científicas sobre consumo de aditivos alimentares por crianças e as possíveis consequências à saúde nessa faixa etária.

**MÉTODOS:**

Revisão de literatura, com busca realizada entre abril de 2020 e abril de 2021 nas bases de dados *Web of Science, Scopus, PubMed* e Google Acadêmico, bem como em sites de órgãos oficiais brasileiros e estrangeiros. Foram selecionados documentos oficiais e estudos publicados a partir do ano 2000. Para a busca, foram utilizados unitermos relacionados a aditivos alimentares, crianças, consumo alimentar e saúde.

**RESULTADOS:**

Aditivos alimentares são substâncias adicionadas intencionalmente aos alimentos para fins tecnológicos. Os alimentos industrializados são as principais fontes de aditivos na alimentação e seu consumo ocorre desde a infância. Observa-se, contudo, que há limitações inerentes ao método científico no que tange à análise de consumo e de toxicidade de aditivos alimentares em humanos, ocasionando escassez de dados na literatura científica. Adicionalmente, dados existentes sugerem que os aditivos apresentam potencial tóxico maior em crianças, considerando que o peso corporal nessa faixa etária é menor em relação ao adulto. Esse contexto ressalta a necessidade de se observar o princípio da precaução, segundo o qual devem-se prevenir os riscos de dano.

**CONCLUSÕES:**

Trata-se de cenário no qual a literatura aponta risco à saúde das pessoas e, em especial, das crianças, cujo dever de proteção deve ser ainda maior, com absoluta prioridade. Assim, pondera-se a relevância de um debate técnico-científico ampliado quanto ao estabelecimento de parâmetros específicos e mais rígidos para crianças, considerando consumo e toxicidade de aditivos, bem como as diversas fontes de exposição a essas substâncias.

## INTRODUÇÃO

Estudos apontam, no Brasil e demais países, um aumento na aquisição de alimentos industrializados para consumo desde os primeiros meses de vida^1,2^, especialmente os classificados como ultraprocessados^a,3^. No Brasil, refrigerantes, bebidas industrializadas à base de frutas (em embalagem longa vida ou em pó), salgadinhos, doces, chocolates, embutidos, pães e biscoitos estão entre os alimentos mais consumidos por crianças^7^. Esses alimentos usualmente contêm quantidades expressivas de açúcar, gordura e sódio^17^, e muitos deles apresentam aditivos alimentares^22^, além de, com frequência, suas embalagens apresentarem estratégias de marketing direcionadas ao público infantil^25^.

Esses aditivos normalmente não são consumidos como alimento nem utilizados como ingrediente típico de alimento e que são adicionadas intencionalmente para fins tecnológicos^26,27^. Os critérios para ingestão e uso dos aditivos nos alimentos industrializados são estabelecidos, mundialmente, pelo *Codex Alimentarius*, programa da Organização das Nações Unidas para Alimentação e Agricultura (FAO) e da Organização Mundial da Saúde (OMS), que desenvolve padrões e diretrizes relacionados a alimentos e estabelece critérios para ingestão e uso de aditivos nos alimentos industrializados, por meio da assessoria de um Comitê Científico Internacional, formado por especialistas de diversos países, chamado *Joint FAO/WHO Expert Committee on Food Additives* (JECFA)^23^.

O comitê de especialistas da FAO/OMS (JECFA) analisa e discute dados de estudos científicos sobre toxicidade e segurança de aditivos e, a partir desses dados, o Comitê estabelece dois valores para cada aditivo alimentar, denominados pelas siglas NOAEL e IDA. O NOAEL, sigla em inglês de *No Observed Adverse Effect Level*, é a quantidade limite em que cada substância não apresentou efeitos tóxicos nos estudos existentes na literatura científica. A partir do valor NOAEL, estipula-se a ingestão diária aceitável (IDA), quantidade estimada que uma substância pode ser consumida diariamente, durante toda a vida, sem apresentar riscos à saúde. Esse valor é calculado pela divisão do valor NOAEL por um coeficiente de segurança/incerteza, estipulado em 100, o qual tem a finalidade de contemplar potenciais incertezas quanto aos dados científicos^23^. Ou seja, considera possíveis diferenças entre modelos animais e humanos, bem como entre sexo e faixas etárias, como toxicidades diferentes para crianças e adultos, por exemplo^28^. Assim, a IDA recomendada pelo *Codex Alimentarius* é, em média, 100 vezes menor do que a quantidade encontrada como segura ou de baixa toxicidade em estudos científicos.

Entretanto, observam-se limitações na avaliação da segurança de consumo de aditivos em seres humanos. Isso porque a maioria dos estudos é realizada em modelos animais ou *in vitro*. Autores ressaltam que as substâncias reagem de maneiras diferentes de acordo com as características celulares de cada organismo^29^. Adicionalmente, os alimentos são considerados misturas complexas de substâncias químicas, nas quais diferentes elementos, de diversos pesos moleculares e configurações químicas, interagem entre si e com o organismo que os ingere^29^. O nível de exposição e a sensibilidade individual são fatores determinantes para avaliar se substâncias como os aditivos têm potencial tóxico^30^.

Tendo em vista que a IDA é estabelecida por quilo de peso, a toxicidade dos aditivos pode ser maior em crianças. Considerando o peso corporal, as crianças bebem mais água, comem mais comida e respiram mais ar do que os adultos. Nos primeiros seis meses de vida, as crianças bebem sete vezes mais água por kg de peso corporal e, com idade entre um e cinco anos, comem de três a quatro vezes mais comida por kg de peso do que um adulto médio^31^. Ademais, como potencialmente têm mais anos de vida futuros do que os adultos, as crianças têm mais tempo para desenvolver doenças crônicas desencadeadas por exposições precoces a substâncias do ambiente^31,32^, como os aditivos alimentares.

Observa-se, contudo, que são escassos na literatura científica estudos experimentais que avaliam a toxicidade de aditivos alimentares em humanos, tanto em adultos como em crianças, o que leva à necessidade de observância do princípio da precaução, segundo o qual deve-se prevenir os riscos de dano. Esse princípio, pela legislação brasileira, tem fundamento no artigo 196 da Constituição, que impõe ao Estado o dever de garantir políticas públicas que visem à redução do risco de doença, além de ações e serviços para a promoção, proteção e recuperação da saúde^33^. Também está implícito no artigo 9 do Código de Defesa do Consumidor, o dever de disponibilizar informação sobre produtos potencialmente nocivos ou perigosos à saúde ou segurança dos consumidores^34,35^, já que o acesso à informação é condição para o exercício consciente da escolha pelos consumidores^36^. O tema tem especial relevância diante do dever de o Estado, a família e a sociedade assegurarem a crianças e adolescentes, com absoluta prioridade, o direito à vida, à saúde, à alimentação etc. conforme preconizado pelo artigo 227 da Constituição Brasileira^33^.

Diante do exposto, não foram encontrados estudos de revisão que busquem discutir os desafios metodológicos envolvidos em pesquisas com humanos sobre consumo de aditivos alimentares e efeitos à saúde em crianças. Dessa forma, o objetivo deste artigo é discutir o contexto das publicações científicas sobre consumo de aditivos alimentares por crianças e as possíveis consequências desse consumo à saúde nessa faixa etária.

## MÉTODOS

Foi realizada revisão narrativa de literatura, que se iniciou com buscas bibliográficas nas bases de dados *Web of Science, Scopus, PubMed* e Google Acadêmico, bem como em sites de órgãos oficiais brasileiros e estrangeiros, entre abril de 2020 e abril de 2021. A estratégia de busca e os conjuntos de unitermos estão expostos na Figura 1.

**Figura 1:** Conjuntos de unitermos e estratégia de busca.

Foram selecionados e analisados os estudos publicados a partir do ano 2000, bem como os documentos oficiais sobre consumo de aditivos alimentares por crianças e consequências à saúde. Além disso, foram utilizados os documentos de recomendações e regulamentações acerca da ingestão e do uso de aditivos em alimentos industrializados.

## RESULTADOS E DISCUSSÃO

### Consumo de Aditivos Alimentares na Infância

Diversas metodologias podem ser utilizadas para estimar o consumo de aditivos alimentares, combinando métodos de avaliação do consumo alimentar e mensuração do conteúdo de aditivos nos alimentos.

Considerando o consumo alimentar, os estudos usualmente utilizam dados de inquéritos populacionais ou aplicam métodos de avaliação do consumo alimentar, especialmente recordatório de 24h, registro alimentar e questionário de frequência alimentar. A partir desses dados, é possível estimar quais alimentos foram consumidos pelos indivíduos e, assim, analisar quais aditivos estavam presentes nesses alimentos^37^.

Como a informação sobre quantidade de aditivos não está disponível no rótulo dos alimentos, alguns estudos utilizam análise laboratorial para identificar a quantidade de aditivos adicionada aos alimentos, sendo a técnica de cromatografia líquida^38,39^ a mais acurada. Outros estudos inferem essa quantidade assumindo que foi adicionado ao alimento, o valor máximo permitido para cada aditivo, estipulado pelo *Codex Alimentarius* (limite máximo) ou pelas agências regulatórias dos países. Essa inferência apresenta mais limitações, posto não haver precisão na determinação dos valores e não ser possível saber se a indústria utilizou a quantidade limite permitida, subestimando ou superestimando a quantidade de aditivo presente no alimento.

Um estudo de revisão^37^ investigou as metodologias empregadas para avaliar o consumo de aditivos no mundo entre os anos de 2000 e 2014. Foram analisados também dados de consumo de aditivos alimentares em todas as faixas etárias. Os estudos encontrados focaram em quatro classes de aditivos: antioxidantes, edulcorantes, corantes e conservantes. A quantificação dos aditivos nos alimentos foi realizada de duas formas: análise laboratorial, usualmente por cromatografia líquida, e estimativa do conteúdo por meio dos limites máximos permitidos, sendo a primeira metodologia a mais frequente nos estudos. O consumo alimentar foi obtido por meio de inquéritos populacionais pela maioria dos estudos, o restante utilizou recordatórios de 24h e questionários de frequência alimentar.

Na revisão, foram avaliados 13 estudos realizados em nove países, grande parte localizados na Europa e na Ásia, que analisaram, prioritariamente na população adulta, o consumo dos edulcorantes sacarina, sucralose, aspartame, estévia, acessulfame e ciclamato. Apenas um estudo realizado no Canadá focou no consumo de edulcorantes por crianças, que não foi superior à IDA. Seis estudos analisaram o consumo de edulcorantes em diversas faixas etárias, incluindo crianças. Desses, um estudo realizado na Índia encontrou valores de consumo de sacarina acima da IDA por crianças e adultos^37^. Nenhum estudo encontrou consumo de aditivos acima da IDA dos antioxidantes BHA^b^, BHT^c^ e TBHQ^d^.

Em 18 estudos foi analisado o consumo de corantes, especialmente tartrazina, amarelo crepúsculo, eritrosina e carmosina. Sete estudos realizados na Índia, um estudo no Kuwait e um na Tailândia encontraram valores de consumo acima da IDA por crianças. Por fim, ao analisarem conservantes como ácido benzoico, ácido sórbico, sulfitos, nitritos e nitratos, os 41 estudos realizados em 26 países encontraram que, em média, a IDA não é excedida. No entanto, nos casos de consumo mais elevado (percentis 90 e 95), a ingestão excedeu a IDA, especialmente nos estudos que analisaram o consumo de conservantes por crianças. Entre as conclusões, os autores apontam a importância e a necessidade de os países terem mecanismos de monitoramento do conteúdo de aditivos utilizados nos alimentos industrializados^37^.

Portanto, observa-se nesse estudo de revisão que o consumo de aditivos por crianças pode exceder os valores de IDA, especialmente para os corantes e conservantes^37^. Relembra-se que esses valores são estipulados por quantidade de aditivo por quilo de peso. Por isso, ressalta-se que esses parâmetros podem ser mais nocivos à saúde de crianças do que de adultos, tendo em vista fatores físicos e biológicos já discutidos. O documento *General Standard on Food Additive*s da FAO e da OMS^26^, principal recomendação sobre o tema em todo o mundo, não traz o valor de quilo de peso a ser considerado como base para o cálculo do limite máximo. Assim, não fica claro se as quantidades consideradas seguras para adição de aditivos nos alimentos consideram o peso infantil e se são realmente seguras para consumo das crianças.

Dados oficiais dos Estados Unidos apontam que o uso de corantes artificiais aumentou, em média, cinco vezes entre 1950 e 2012, de 12 mg para 68 mg per capita por dia^40^. No Brasil, autores analisaram em laboratório a quantidade de corantes artificiais presentes em quatro tipos de alimentos: bala, goma de mascar, confeito de chocolate e cereais matinais e os resultados mostraram que os corantes tartrazina e amarelo crepúsculo foram os mais utilizados. As balas e os confeitos de chocolate apresentaram quantidades de corantes dentro do permitido. Contudo, 33% das gomas de mascar tinham quantidades de corantes artificiais acima das permitidas pela Anvisa, sendo que uma das marcas superou em cinco vezes o limite máximo estipulado. Já todas as amostras de cereais matinais analisadas apresentaram quantidades acima das permitidas para corantes artificiais^41^. Ressalta-se que os alimentos analisados são frequentemente consumidos pela população desde a infância. Além disso, os corantes artificiais, em especial o amarelo crepúsculo e o tartrazina, são alvo de estudos toxicológicos que os relacionam ao desenvolvimento de sintomas de alergia e hiperatividade em crianças^42^.

Ademais, questiona-se quanto ao risco decorrente do consumo cumulativo de aditivos, advindos de diversos tipos de alimentos ingeridos ao longo do dia. Utilizando como exemplo os alimentos analisados pelo estudo^41^, é possível inferir que o risco de toxicidade por corantes parece ser elevado quando uma criança consome no café da manhã um cereal matinal e uma ou mais gomas de mascar durante o dia. Considerando os demais alimentos consumidos a cada dia e os outros aditivos alimentares ingeridos, o risco de toxicidade torna-se maior.

Nesse sentido, reforça-se que as crianças são mais vulneráveis ao consumo de aditivos alimentares. Conforme evidenciado pelo estudo de revisão^37^, os resultados sobre consumo de aditivos alimentares divergem consideravelmente, dependendo do país, da substância analisada e da metodologia empregada, contudo, quando analisando o consumo de aditivos por crianças, os valores da IDA são frequentemente ultrapassados.

No Quadro 1 são apresentados os principais resultados de estudos encontrados que avaliaram o consumo de aditivos alimentares por crianças.

**Quadro 1:** Estudos que avaliaram o consumo de aditivos alimentares por crianças, em ordem cronológica

Autor, ano	País	Avaliação do consumo	Estimativa do conteúdo de aditivo	Aditivos avaliados	Resultados
Husain et al.^45^ (2007)	Kuwait	Recordatório alimentar de 24h.	Análise laboratorial de 344 amostras de alimentos.	Corantes artificiais: tartrazina, amarelo crepúsculo, carmosina, vermelho allura, alaranjado G, eritrozina, verde rápido, índigo-carmim, azul brilhante, negro brilhante, marrom chocolate HT.	Consumo por 3.141 crianças acima de 5 anos. Em 4 corantes analisados o consumo foi de 2 a 8 vezes acima da IDA (tartrazina, amarelo crepúsculo, carmosina e vermelho allura).
Schumann et al.^46^ (2008)	Brasil	Questionário quali-quantitativo de frequência alimentar de sucos em pó, pó para gelatina e refrigerante.	Inferência pelo limite máximo permitido.	Corantes artificiais: amarelo crepúsculo, amaranto e tartrazina.	Consumo por 150 crianças até 10 anos. O consumo dos corantes amarelo crepúsculo e amaranto podem estar excedendo a IDA em 20% e 90% das crianças, respectivamente.
Sardi et al.^47^ (2010)	Suíça	Dados de compra fornecidas pelo cartão de uma rede varejista e entrevistas.	Cartão de uma rede varejista, que contém os dados de composição dos alimentos.	Corante amarelo crepúsculo.	Amostra representativa da população com 2.390 indivíduos de todas as faixas etárias. Na faixa etária de 1 a 10 anos a média de consumo e os valores máximos foram acima da IDA.
Dixit et al.^48^ (2011)	Índia	Questionário de frequência alimentar.	Análise laboratorial	Corantes artificiais: amarelo crepúsculo, eritrosina, tartrazina, carmosina, ponceau 4R e azul brilhante.	Consumo por 245 indivíduos de 4 a 18 anos. Considerando os valores médios de consumo, a eritrozina excedeu o valor da IDA. Considerando os valores máximos de consumo (percentil 95), além da eritrozina, o amarelo crepúsculo também excedeu a IDA.
Larsson et al.^49^ (2011)	Suécia	Dados oficiais do governo, obtidos por diário alimentar de 4 dias.	Dados oficiais do governo, obtidos por análise laboratorial.	Conservantes nitritos e nitratos.	Consumo por 2.259 crianças abaixo da IDA. Entretanto, considerando a conversão endógena de nitrato em nitrito, 12% das crianças de 4 anos podem exceder a IDA.
Lok et al.^50^ (2011)	China	Questionário de frequência alimentar.	Análise laboratorial de 87 alimentos.	11 corantes artificiais: tartrazina, amarelo de quinoleína, amarelo crepúsculo, amaranto, vermelho “*Chromotrope FB*”, ponceau 4R, vermelho allura, eritrozina, índigo-carmim, azul brilhante, lissamina “*Green B*”.	Consumo por 142 crianças de 8 e 9 anos. O consumo médio de corante amarelo crepúsculo foi superior à IDA por menino de 9 anos. O consumo dos demais corantes não excedeu a IDA.
Polônio e Peres^14^ (2012)	Brasil	Questionário de frequência alimentar e recordatório 24h.	Inferência pelo limite máximo permitido.	Corantes artificiais: amarelo crepúsculo, azul brilhante, amaranto, eritrozina, tartrazina, vermelho 40.	Questionário aplicado em 148 mães de crianças entre 3 e 5 anos. O consumo dos corantes vermelho bordô e amarelo crepúsculo podem estar excedendo a IDA em 56% e 25% das crianças, respectivamente.
Urtiaga et al.^51^ (2013)	Espanha	Inquérito populacional.	Análise laboratorial de 909 alimentos.	Antioxidantes sulfitos.	Consumo de 1.055 indivíduos de 4 a 18 anos. O consumo foi superior à IDA em 4% das crianças.
Vin et al.^52^ (2013)	Itália, França, Irlanda e Reino Unido	Inquéritos populacionais.	Inferência pelo limite máximo permitido e dados fornecidos pela indústria.	13 aditivos: benzoatos, nitritos, sulfitos, hidroxitolueno butilado, polissorbatos, ésteres de sucroses, sucroglicerídeos, ésteres de poliglicerol de ácidos graxos, estearoil-lactilatos, ésteres de sorbitano, fosfatos, aspartame e acessulfame.	Consumo por 16.603 indivíduos de todas as faixas etárias. Considerando os consumos acima do percentil 95, 4 dos 13 aditivos apresentaram consumo acima da IDA em crianças (sulfitos, polissorbatos, estearoil-lactilatos e ésteres de sorbitano).
Diouf et al.^53^ (2014)	Alemanha	Inquéritos populacionais.	Inferência pelo limite máximo permitido	Corantes: carmim, amarelo crepúsculo, ponceau 4R, vermelho allura e colorau.	Consumo por 1.234 crianças de 6 a 11 anos e 1.272 adolescentes de 12 a 17 anos. Em média, o consumo de ponceu 4R excedeu a IDA. Considerando os consumos máximos (acima do percentil 95), o consumo de amarelo crepúsculo e ponceu 4R excederam a IDA.
Mancini et al.^54^ (2015)	França	Inquéritos populacionais em crianças menores de 3 anos.	Inferência pelo limite máximo permitido.	Conservantes: benzoatos, parabenos, nitritos, nitratos; Antioxidantes: BHA e BHT; Edulcorante: aspartame.	Consumo por 706 crianças de 0 a 3 anos. O consumo de benzoatos, nitritos e BHA excederam a IDA em, respectivamente, 25%, 54% e 20% da população estudada.
Suomi et al.^55^ (2016)	Finlândia	Dados oficiais do governo, obtidos por diário alimentar de 3 dias.	Dados oficiais do governo, obtidos por análise laboratorial.	Conservantes: nitritos e nitratos em carnes curadas e na água.	Consumo por 1.471 crianças de 1, 3 e 6 anos. Consumo abaixo da IDA para todas as faixas etárias.
Reddy et al.^56^ (2015)	Índia	Recordatório 24h.	Análise laboratorial.	Conservantes: benzoato de sódio e sorbato de potássio.	Consumo por 960 indivíduos de 2 a 19 anos. Consumo abaixo da IDA.
Martyn et al.^57^ (2016)	Irlanda	Dados oficiais do governo.	Dados oficiais do governo, obtidos por análise laboratorial.	Edulcorantes: acessulfame K, sacarina, aspartame e sucralose.	Consumo por 500 crianças de 1 a 4 anos. Consumo abaixo da IDA.
Feitosa et al.^58^ (2017)	Brasil	Dados oficiais do governo.	Inferência pelo limite máximo permitido.	Corante amarelo crepúsculo.	Consumo por amostra representativa da população acima de 10 anos. Consumo acima da IDA para as crianças acima de 10 anos, considerando a prevalência de consumo dos alimentos analisados.
Bastaki et al.^59^ (2017)	EUA	Dados oficiais do governo.	Dados fornecidos pela indústria.	Corantes: vermelho allura, tartrazina, azul brilhante, amarelo crepúsculo, índigo-carmim, eritrozina, verde rápido.	Consumo por 16.011 indivíduos de todas as faixas etárias. Consumo abaixo da IDA, para todas as faixas etárias.
Choi e Suh^60^ (2017)	Coréia	Inquérito populacional.	Análise laboratorial de 287 alimentos.	Conservante nitrito.	Consumo por 8.019 indivíduos de todas as faixas etárias. Consumo médio abaixo da IDA.
Martyn et al.^61^ (2017)	Brasil, México, Canadá, EUA	Inquéritos populacionais dos 4 países.	Dados fornecidos pela indústria.	Conservantes benzoatos em bebidas não alcóolicas.	Consumo por amostra representativa da população dos 4 países. Consumo pode exceder a IDA acima do percentil 95 no Canadá e no México.
Bel et al.^62^ (2018)	Bélgica	Dados oficiais do governo.	Inferência pelo limite máximo permitido e análise laboratorial.	Emulsificantes: estearoil-2-lactilato de sódio e de cálcio.	Consumo por amostra representativa da população de todas as faixas etárias. Limite máximo: consumo de 92% das crianças possivelmente excede a IDA; Análise laboratorial: consumo excedeu a IDA em 1,9% das crianças.
Garavaglia et al.^63^ (2018)	Argentina	Inquérito populacional.	Rótulo dos alimentos e dados fornecidos pela indústria.	Edulcorantes: acessulfame K, sacarina, aspartame, ciclamato e sucralose.	Consumo por 2.664 indivíduos de 2 a 18 anos. Consumo médio abaixo da IDA. Considerando os consumos máximos, 0,3% das crianças excederam a IDA para sacarina e 0,9% para ciclamato.
Long et al.^39^ (2019)	Vietnã	Recordatório 24h.	Análise laboratorial.	Conservantes: benzoatos e sorbatos; Edulcorantes: ciclamato e sacarina; Corantes: tartrazina e amarelo crepúsculo.	Consumo por 10.499 indivíduos de todas as faixas etárias. O consumo de benzoatos foi superior à IDA em 4,6% das crianças menores de 5 anos e 2,6% das crianças entre 6 e 10 anos.
Martínez et al.^64^ (2020)	Chile	Questionário de frequência alimentar.	Rótulo dos alimentos e dados fornecidos pela indústria.	Edulcorantes: acessulfame K, estévia, sacarina, aspartame, ciclamato e sucralose.	Consumo por 250 crianças entre 6 e 12 anos. Consumo abaixo da IDA. Entretanto, todas as 250 crianças avaliadas consumiram diariamente pelo menos um tipo de edulcorante.

IDA: ingestão diária aceitável; BHA: do inglês *buthylated hydroxyanisole*; BHT: do inglês *butylated hydroxytoluene*.

Foram encontrados 22 estudos, realizados em 21 países, que analisaram o consumo de seis classes funcionais de aditivos por crianças: corantes, conservantes, edulcorantes, antioxidantes, emulsificantes e estabilizantes. Destaca-se que os corantes, em especial o amarelo crepúsculo e a tartrazina, foram os mais estudados. Em dezesseis estudos, pelo menos um aditivo teve consumo estimado como acima dos limites de segurança, das seguintes classes funcionais: corantes^14,45^, conservantes^39,49,54,61^, antioxidantes^51,52,54^, emulsificantes^62^ e edulcorantes^63^.

Em 13 estudos o consumo alimentar foi analisado por meio de inquéritos populacionais previamente coletados. Dados oficiais governamentais, questionário de frequência alimentar, recordatório de 24h e informações de compra em redes de supermercados também foram utilizados. Em 10 estudos os aditivos foram quantificados por meio de análise laboratorial e o restante inferiu as quantidades pelo limite máximo permitido (sete estudos) e informações fornecidas pela indústria (cinco estudos).

Encontrou-se quatro estudos no Brasil: um sobre conservantes (benzoatos) e três sobre corantes. Martyn et al.^61^ (2017) utilizaram dados de consumo oriundos da Pesquisa de Orçamentos Familiares (POF) 2008/2009 do Instituto Brasileiro de Geografia e Estatística (IBGE), com 34.003 indivíduos acima de 10 anos, e não identificaram valores acima da IDA para os benzoatos, quantificados a partir de contato feito com fabricantes. Entretanto, Schumann et al.^46^ (2008), Polônio e Peres^14^ (2012), bem como Feitosa et al.^58^ (2017), encontraram valores de consumo acima da IDA para os corantes amarelo crepúsculo, vermelho bordô e amaranto. Esses três estudos estimaram a quantidade de aditivos nos alimentos por meio dos limites máximos permitidos. Contudo, enquanto Schumann et al.^46^ (2008) e Polônio e Peres^14^ (2012) aplicaram questionário de frequência alimentar com as crianças participantes, Feitosa et al.^58^ (2017) utilizaram dados de consumo da POF/IBGE 2008/2009.

Assim como no Brasil, o consumo de corantes parece exceder a IDA no Kuwait^45^, na Suíça^47^, na Índia^48^, na China^50^ e na Alemanha^53^. Na análise do consumo de conservantes observa-se que há resultados apontando consumo acima da IDA e abaixo dela. Ao contrário do Brasil^61^, no Vietnã^39^, no Canadá^61^, no México^61^ e na França^54^, o consumo de conservantes excedeu a IDA. Com relação às demais classes de aditivos estudadas, há evidências de possíveis consumos excessivos de antioxidantes^51,54^ e emulsificantes^62^ por crianças.

A partir dos dados disponíveis na literatura científica, resumidos no Quadro 1, ressalta-se um possível consumo elevado de aditivos na infância, de acordo com os valores de IDA estipulados pelo *Codex Alimentarius*, especialmente de corantes. Adicionalmente, ressalta-se que não foi encontrado nenhum artigo que analisasse o consumo cumulativo por crianças de diferentes aditivos ao longo do tempo. Essa lacuna na literatura científica é relevante, tendo em vista que, ao longo de cada dia, os indivíduos consomem múltiplas porções de diferentes tipos de alimentos que podem, potencialmente, ser fontes de diversos aditivos.

Corroborando essa afirmação, um dos objetivos do estudo de coorte NutriNet-Santé, realizado na França com 106 mil adultos, foi descrever os perfis de exposição a diferentes aditivos pela população (um aditivo ou misturas de diferentes tipos de aditivos). Foram encontrados cinco grupos, compostos por alimentos distintos. O primeiro grupo é composto pelos aditivos encontrados em biscoitos e bolos (lecitinas, mono e diglicerídeos de ácidos graxos, carbonatos, difosfatos, glicerol e sorbitol), consumidos majoritariamente por indivíduos com pós-graduação, não-fumantes, com as maiores médias de consumo calórico e lipídico. O segundo grupo corresponde aos aditivos encontrados em caldos, manteiga, pães e substitutos de refeições (amidos modificados, glutamato monossódico, ésteres de ácidos graxos e BHA), consumidos por indivíduos mais velhos, fisicamente ativos e não-fumantes. Em seguida, o terceiro grupo de aditivos encontrados em sobremesas lácteas, cereais matinais e produtos de panificação (carragena, ácido láctico, propionato de cálcio e fosfatos), consumidos por pessoas com os maiores consumos de carboidratos. Já o quarto grupo diz respeito aos aditivos encontrados em molhos e carnes processadas (nitrito de sódio, eritorbato de sódio, fosfatos e cochonilha), frequentemente consumidos por homens com os menores níveis de escolaridade. Por fim, o quinto grupo se refere aos aditivos encontrados em bebidas açucaradas e adoçadas artificialmente (mistura de edulcorantes – acessulfame K, aspartame, sucralose, glicosídeos de esteviol – corantes, pectinas, carotenos, citrato de sódio, benzoatos, fosfatos, nitratos), consumidos pelos indivíduos mais jovens, com os maiores índices de massa corporal, menores níveis de atividade física e mais propensos a serem fumantes^65^.

Em contrapartida, esse estudo destaca um sexto grupo, relativo aos menores consumos de aditivos, encontrados em alimentos integrais, leguminosas, cereais matinais sem adição de açúcar, sucos de vegetais, oleaginosas, óleos vegetais e queijos. Esse grupo de alimentos foi consumido majoritariamente por mulheres, com os menores consumos calóricos e de alimentos ultraprocessados e maiores consumos de alimentos orgânicos e bebidas alcóolicas. Como conclusões, os autores destacam que o impacto na saúde e os efeitos potenciais do consumo de diferentes tipos de aditivos devem ser explorados em estudos epidemiológicos e experimentais. Seguindo o princípio da precaução, várias autoridades de saúde pública em todo o mundo começaram recentemente a recomendar o consumo de alimentos sem ou com o mínimo possível de aditivos^65^.

Observou-se que os estudos apresentados no Quadro 1 analisam o consumo alimentar e quantificam os aditivos nos alimentos utilizando metodologias distintas. Os principais métodos de avaliação do consumo alimentar foram recordatório de 24h, dados de aquisição de alimentos e questionário de frequência alimentar. Já a quantidade de aditivos nos alimentos foi identificada por análise laboratorial, dados fornecidos pela indústria ou inferida por meio de estimativa por limite máximo. Esse cenário de pouca padronização no método de coleta e análise dos dados pode indicar uma limitação metodológica nos estudos sobre o tema, na medida em que os resultados dos estudos não podem ser comparáveis entre si, fragilizando as evidências científicas existentes sobre o consumo de aditivos.

Ressalta-se que há aditivos que não apresentam valores de limite máximo determinados por não haver uma IDA estabelecida pelo JECFA e, portanto, seu uso é recomendado pelo *Codex Alimentarius* e/ou autorizado pelas agências regulatórias dos países na base de *quantum satis*. Esse termo significa que é permitido ao fabricante adicionar a quantidade de aditivo que entender necessária e suficiente para atingir a função tecnológica pretendida, não havendo um valor máximo determinado para adição no momento da fabricação (limite máximo). Como exemplo, no Brasil a Anvisa autoriza em produtos de panificação e bolos, entre outros aditivos, o uso do emulsificante lecitina de soja e de todos os aromatizantes na quantidade *quantum satis*^66^. Com isso, infere-se que somente é possível analisar o efetivo consumo desses aditivos por meio da análise laboratorial ou contato com a indústria, limitando a realização de estudos sobre tais substâncias.

Contudo, embora o limite *quantum satis* seja autorizado no Brasil e validado pelo *Codex Alimentarius*, conjectura-se quanto à subjetividade da definição dessa quantidade de aditivo a ser adicionado aos alimentos, bem como aos potenciais riscos, entendendo-se que o fabricante é autorizado a adicionar a quantidade de aditivo que considerar necessária, sem necessariamente ponderar quanto à segurança de consumo da substância. Ademais, ressalta-se que o consumidor não tem nenhum mecanismo de acesso à informação nem quanto à quantidade permitida de uso nem quanto à quantidade realmente adicionada ao alimento. Isso ocorre, pois, a legislação em vigor indica que os aditivos alimentares devem ser declarados depois dos ingredientes (e não em ordem decrescente, da respectiva proporção, como acontece com os ingredientes).

Para além da falta de informação ao consumidor nos rótulos, esse contexto pode, portanto, ocasionar imprecisões na quantificação do consumo de aditivos. Consequentemente, pode trazer limitações não somente para a análise do consumo de aditivos, mas também para a avaliação de toxicidade e efeitos à saúde em humanos.

### Aditivos Alimentares e Consequências à Saúde de Crianças

O consumo de alimentos industrializados ultraprocessados pode estar diretamente relacionado ao desenvolvimento de obesidade, diabetes, câncer e demais doenças crônicas não transmissíveis^67,68^. Contudo, ainda é incerto quais as variáveis presentes nos alimentos ultraprocessados que mais contribuem para esses resultados, citando a necessidade de analisar melhor os aditivos alimentares, dentre outros componentes^69^.

A maior parte dos estudos para identificação da toxicidade dos aditivos alimentares é realizada com roedores em laboratório^70^, os quais devem seguir protocolos de elaboração e execução para que tenham seus resultados validados pelo *Codex Alimentarius* e por agências regulatórias de todo o mundo ao estabelecerem a IDA. O principal protocolo utilizado advém das diretrizes da *Organization for Economic Cooperation and Development* (OECD) para testes de produtos químicos, que reúnem cerca de 150 métodos, acordados internacionalmente, para identificar e caracterizar os perigos potenciais dos produtos químicos^85^. Embora existam protocolos metodológicos rígidos para os estudos experimentais, questiona-se, à luz da metodologia científica e do princípio da precaução, se é adequado extrapolar para seres humanos resultados encontrados em células ou modelos animais, visando estabelecer limites de consumo humano para substâncias potencialmente tóxicas, como os aditivos.

No Informe Técnico nº 70 de 2016^86^, que objetivou esclarecer questões sobre a declaração e alegações de conteúdo para aditivos alimentares na rotulagem de alimentos, a Anvisa expõe que:

Apesar de os aditivos alimentares serem submetidos a uma avaliação de segurança e de eficácia tecnológica previamente à autorização de uso, a abordagem mundialmente aceita e utilizada na avaliação de segurança possui diversas limitações, tais como: a dificuldade na transposição de dados toxicológicos obtidos em estudos com animais para humanos e a dificuldade de previsão da variabilidade interindividual. Ademais, novos estudos têm sugerido que estas substâncias podem causar reações adversas não identificadas na avaliação de segurança, incluindo reações de alergia, intolerâncias alimentares e hiperatividade (...)

Entretanto, são esses estudos de avaliação toxicológica, realizados principalmente em modelos animais, que embasam o *Codex Alimentarius* nas recomendações de IDA e limites máximos nos alimentos. Considera-se esse aspecto uma limitação metodológica nos estudos sobre efeitos à saúde em humanos, uma vez que, mesmo adequadamente executados, os efeitos (ou ausência deles) encontrados em animais não necessariamente ocorrerão em humanos ou na mesma intensidade.

Dybing et al.^29^ (2002)ressaltam que, embora metodologicamente existam fórmulas para extrapolar os resultados para seres humanos, sabe-se que as substâncias reagem de maneiras diferentes de acordo com as características celulares de cada organismo. Além disso, autores questionam o uso do valor NOAEL como referência para embasar as recomendações de IDA para aditivos. O tamanho amostral dos estudos é considerado ponto sensível, por variarem entre eles e por, muitas vezes, haver amostras pequenas para considerar que uma substância não apresenta efeitos tóxicos^87^. Ademais, ponderam que a determinação do valor NOAEL não considera a progressão do efeito tóxico em relação à duração e/ou dose do aditivo^88^.

A título de exemplo quanto às controvérsias e desafios metodológicos para atestar toxicidade de aditivos alimentares, destaca-se a recente discussão quanto à utilização de dióxido de titânio em alimentos. O uso desse corante é atestado pelo JECFA no limite *quantum satis* desde 1969, ano da última avaliação toxicológica realizada pelo comitê. Nessa análise, os estudos não demonstraram efeitos tóxicos do dióxido de titânio em modelos animais. Entretanto, em março de 2021, a agência reguladora da União Europeia (*European Food Safety Authority* – EFSA) publicou uma nova avaliação de toxicidade e concluiu que o aditivo não deve mais ser considerado seguro para consumo humano, em nenhuma quantidade^89^. Esse debate se iniciou a partir de um posicionamento da agência reguladora da França (*l’Agence nationale de sécurité sanitaire de l’alimentation* – ANSES) que, após análise realizada por especialistas, publicou um decreto suspendendo a comercialização de alimentos que contém dióxido de titânio, a partir de 1º de janeiro de 2021, por não considerar esse aditivo seguro para consumo humano^90^. Assim, o uso do dióxido de titânio vem sendo (re)discutido em diversos países do mundo, entrando também na Agenda Regulatória de Alimentos 2021/2023 da Anvisa^91^.

Nesse contexto, as avaliações de segurança e toxicidade são geralmente realizadas com apenas um aditivo, subestimando os efeitos de associar duas ou mais substâncias, que podem interagir ao serem ingeridas^92^. Assim, o consumo cumulativo e concomitante de diferentes tipos de aditivos é outro aspecto latente no que tange à toxicidade. É pouco estudada a interação de diferentes aditivos conjuntamente, tanto entre si quanto com o organismo humano. Como consequência, é incerta a relevância desse assunto no estabelecimento da IDA dos aditivos alimentares. Em estudo realizado em 50 ratos Wistar, por exemplo, foi avaliado o efeito do consumo concomitante de diferentes tipos de aditivos (corantes, conservantes e edulcorantes) em marcadores sanguíneos e nos tecidos do fígado, rim e cérebro. Foram escolhidos os aditivos presentes em alimentos consumidos por crianças e que fossem alvo de controvérsias quanto à segurança de consumo. Como resultado, os autores pontuam que, embora o valor NOAEL estabelecido para cada aditivo separadamente pareça ser seguro, quando diferentes tipos de aditivos são consumidos conjuntamente essa segurança pode ser comprometida. O consumo de diferentes tipos de conservantes e, concomitantemente, de conservantes, corantes e edulcorantes demonstraram riscos potenciais de danos ao DNA de células cerebrais, renais e hepáticas. Além disso, à medida que o número de aditivos administrados aumentava, houve redução dos níveis de hemoglobina, albumina e proteína sérica total, bem como um aumento da ureia, creatinina, bilirrubina e atividade enzimática do fígado. Essas alterações podem desencadear diversos danos metabólicos, bem como doenças decorrentes dos danos ao DNA e dos desequilíbrios nos parâmetros bioquímicos^83^.

Na Figura 2 são apresentados os resultados associando toxicidade de aditivos alimentares em modelos animais^70^.

**Figura 2:** Doenças identificadas em modelos animais decorrentes do consumo de aditivos alimentares.

Um estudo de revisão sistemática^93^ sobre os potenciais riscos dos conservantes benzoatos e sorbatos apontou que, isoladamente, essas substâncias parecem não apresentar efeitos tóxicos em mamíferos. Entretanto, em contato com outros aditivos no ambiente gástrico, como os nitritos e o ácido ascórbico, podem formar substâncias com potencial carcinogênico. Além disso, os resultados em modelos animais indicam potenciais efeitos teratogênicos e lesões hepáticas; efeitos deletérios ao desenvolvimento neuronal e retardo do crescimento, anormalidade hematológica e lesão de órgãos. Os autores da revisão discutem, ainda, que nos estudos realizados *in vitro*, há resultados indicando aumento do estresse oxidativo, danos ao material genético, inibição na liberação de leptina nos adipócitos e danos mitocondriais^93^.

Em seres humanos, não são conclusivos a maioria dos estudos de revisão encontrados que avaliaram as possíveis consequências do consumo de aditivos à saúde. Possivelmente, resultados inconclusivos ocorrem pela impossibilidade de comparação determinada pelas diferentes metodologias utilizadas. Nesse sentido, destacam-se estudos que avaliaram o consumo de edulcorantes artificiais e efeitos metabólicos^94,95^, bem como nitritos e nitratos e câncer^96,97^. Esses estudos apontam a necessidade de mais investigações em seres humanos, para traçar resultados conclusivos.

Uma revisão de escopo com o objetivo de mapear possíveis desfechos de saúde associados ao consumo frequente de edulcorantes artificiais encontrou 372 artigos que investigaram diversas alterações de saúde, como: câncer, diabetes, alterações no apetite, cárie, ganho de peso, obesidade, dores de cabeça, depressão, efeitos comportamentais e cognitivos, efeitos neurológicos, risco de parto prematuro, efeitos cardiovasculares e risco de doença renal crônica. Entretanto, os autores consideram que os resultados são inconclusivos e apontam a necessidade de mais pesquisas, especialmente estudos longitudinais com procedimentos metodológicos rigorosos e detalhados, bem como revisões sistemáticas bem executadas, permitindo resumo quantitativo e análise de validade dos dados existentes^98^.

Outros estudos de revisão pontuam que, embora não haja uma conclusão que confirme a relação entre consumo de aditivos e desfechos de saúde, também não há evidências para rejeitá-la^99^. Sabe-se que a metodologia dos estudos experimentais e observacionais deve ser cuidadosamente analisada para verificar se houve rigor metodológico que valide cientificamente os resultados. Entretanto, os dados obtidos em estudos primários devem ser considerados, especialmente por órgãos de saúde pública e agências reguladoras. Adicionalmente, face à falta de consenso na literatura científica quanto aos danos à saúde, especialmente para substâncias potencialmente tóxicas, como os aditivos alimentares, deve-se considerar o princípio da precaução, que prevê, quando não há comprovação científica de segurança, a adoção de medidas contra riscos potenciais, sempre que houver perigo de dano grave ou irreversível^102^.

Embora muitos estudos não encontrem resultados conclusivos, há na literatura científica revisões que, ao avaliarem estudos primários experimentais e/ou observacionais, apontam a relação entre consumo de aditivos por humanos e potenciais danos à saúde. Face à escassez de estudos de revisão com esse objetivo, o Quadro 2 resume os resultados encontrados, tanto em crianças quanto em adultos.

**Quadro 2:** Estudos de revisão, com resultados conclusivos, que analisaram os efeitos do consumo de aditivos à saúde de adultos e crianças

Autores/ano	Metodologia	Aditivos	Efeitos à saúde
**Adultos**			
Vally et al.^103^ (2009)	Revisão de literatura	Sulfitos	Problemas respiratórios
Song et al.^104^ (2015)	Metanálise que incluiu 22 artigos consistindo em 49 estudos - 19 estudos para nitratos, 19 estudos para nitritos e 11 estudos para *N*-nitrosodimetilamina.	Nitratos, nitritos e nitrosaminas.	Desenvolvimento de câncer gástrico.
Romo-Romo et al.^105^ (2016)	Revisão sistemática que incluiu 14 estudos observacionais e 28 experimentais. Metanálise com dois estudos experimentais.	Edulcorantes	Desregulação metabólica.
Paula Neto et al.^92^ (2017)	Revisão de literatura.	Citrato, edulcorantes artificiais, carragena, emulsificantes.	Alteração na microbiota e desregulação metabólica.
Azad et al.^106^ (2017)	Revisão sistemática com metanálise que incluiu 7 estudos experimentais e 30 estudos de coorte.	Edulcorantes	Ganho de peso e efeito cardiometabólico.
Crowe et al.^107^ (2019)	Revisão de literatura	Nitrito de sódio	Desenvolvimento de câncer colorretal.
**Crianças**			
Schab e Trinh^42^ (2004)	Revisão sistemática com metanálise que incluiu 15 estudos experimentais com crianças.	Corantes artificiais	TDAH
Polônio e Peres^43^ (2009)	Revisão sistemática que incluiu 13 estudos transversais e experimentais com crianças.	Corantes artificiais	Rinite, urticária e angioedema.
Kanarek^44^ (2011)	Revisão de literatura de estudos experimentais com crianças.	Corantes artificiais	TDAH

TDAH: transtorno de déficit de atenção e hiperatividade.

Foram encontrados nove estudos, dos quais seis analisaram os efeitos à saúde em adultos^92,103^ e três em crianças^42^. Os resultados parecem apontar para o potencial de desenvolvimento de transtorno de déficit de atenção e hiperatividade (TDAH), alterações na microbiota intestinal, desregulação metabólica, ganho de peso, efeitos cardiometabólicos, desenvolvimento de câncer no trato gastrintestinal, problemas respiratórios, rinite, urticária e angioedema. Além disso, os estudos analisaram os efeitos à saúde de diferentes tipos de aditivos, como: sulfitos, nitritos, nitratos, nitrosaminas, edulcorantes, carragena, citrato e emulsificantes.

Em crianças, os estudos de revisão identificados analisaram os efeitos à saúde ocasionados por apenas uma classe funcional de aditivos, os corantes. Foram encontrados três estudos, associando seu consumo a efeitos em curto e longo prazo no desenvolvimento de reações alérgicas, como rinite, urticária e angioedema, bem como transtornos comportamentais, como o TDAH. Schab e Trinh^42^ (2004) apontam que os corantes artificiais promovem a hiperatividade em crianças, considerando sintomas medidos por escalas de avaliação comportamental. Kanarek^44^ (2011), ao analisar as mesmas variáveis, destaca que, embora o consumo de corantes pareça estar associado ao agravamento dos sintomas de hiperatividade e/ou déficit de atenção, a retirada completa deles da alimentação pode não ser suficiente para o tratamento dos sintomas do TDAH, considerando a multifatorialidade das causas.

Polônio e Peres^43^ (2009), destacam que o número de estudos foi maior e os resultados mais consistentes quanto às manifestações clínicas de hipersensibilidade não específica, como rinite, urticária e angioedema, relacionadas ao consumo de corantes artificiais. Entretanto, apontam também que, embora com resultados divergentes, estudos encontraram relação entre o consumo de aditivos e o desenvolvimento de cânceres, especialmente quando o consumo foi superior à IDA.

Embora não se tenha encontrado nenhum estudo de revisão com resultados conclusivos acerca dos efeitos dos edulcorantes à saúde de crianças, Shum e Georgia^108^ (2021), em sua revisão, ressaltam que o consumo desse aditivo parece ser frequente nessa faixa etária e, por vezes, superior aos limites recomendados. Assim, apontam a necessidade de estudos quanto aos potenciais efeitos à saúde de crianças, especialmente quanto ao possível risco de desenvolvimento de diabetes tipo 2 e de doenças cardiometabólicas decorrentes do consumo de edulcorantes; pontuam ainda a importância de investigar como a exposição intrauterina a edulcorantes pode influenciar resultados metabólicos durante a vida.

Nesse sentido, uma revisão sistemática com metanálise analisou os efeitos do consumo materno de edulcorantes em desfechos durante o nascimento, especificamente o peso ao nascer, o parto prematuro e a idade gestacional. Os autores ressaltam que as evidências são de baixa qualidade, contudo, sugerem que o consumo diário de edulcorantes durante a gravidez esteja associado ao risco aumentado de parto prematuro, diminuindo a idade gestacional e aumento do peso ao nascer^109^.

Há poucos estudos experimentais relacionando os efeitos do consumo de aditivos à saúde de crianças, mas há hipóteses em estudo. A pesquisa mais citada e que trouxe resultados mais robustos até o momento foi realizada na Inglaterra e publicada em 2007, na revista *Lancet*, por McCann et al.^110^ (2007). Trata-se de um ensaio clínico randomizado, placebo controlado e duplo-cego. Os autores avaliaram o efeito do consumo de duas bebidas contendo diferentes concentrações de aditivos alimentares, em comparação ao placebo, em desfechos comportamentais de hiperatividade em crianças de três a quatro anos e de oito a nove anos. As duas bebidas continham corantes artificiais (amarelo crepúsculo, carmosina, tartrazina e ponceau 4R), em maior concentração na segunda bebida, e conservante benzoato de sódio na mesma concentração nas duas bebidas. Como resultado, o consumo das duas bebidas, nas duas faixas etárias, aumentou o nível médio de hiperatividade em relação ao placebo, correlacionando o consumo de corantes artificiais e de benzoato de sódio ao desfecho comportamental em crianças^110^. Ressalta-se que houve críticas à metodologia do estudo, especialmente à dose de aditivos empregada^111^. Contudo, McCann et al.^110^ (2007) indicam no método do artigo que as doses empregadas nas bebidas para crianças de três a quatro anos correspondem ao consumo de dois pacotes de bala de 56g. Já as quantidades de aditivos presentes em uma das bebidas das crianças de oito e nove anos correspondem a quatro pacotes de bala. Questiona-se, inicialmente, qual a criança dentro das faixas etárias pesquisadas que consumiria habitualmente essa quantidade de balas?

Sabe-se que ensaios clínicos randomizados fornecem altos níveis de evidência científica, se adequadamente executados. Além disso, esse delineamento de estudo costuma ter espaço de publicação em revistas de alto fator de impacto, como ocorreu no estudo supracitado^110^. Entretanto, considera-se relevante a discussão acerca de aspectos éticos envolvidos no delineamento e execução desse tipo de estudo, quando o desfecho principal é o efeito da ingestão de substâncias potencialmente nocivas ao organismo.

Tal situação é ainda mais latente quando o alvo dos estudos são crianças. Primeiramente, é necessária autorização dos responsáveis para participação de crianças em qualquer tipo de estudo. Questiona-se se todos os riscos envolvidos na administração de substâncias potencialmente tóxicas, como os aditivos, são plenamente esclarecidos aos responsáveis para que autorizem a participação de uma criança em um estudo com esse delineamento, em que há riscos envolvidos e, certamente, a criança não terá qualquer benefício à saúde e ao bem-estar ao participar. Além disso, pondera-se quanto aos efeitos nocivos e permanentes que podem ser gerados aos participantes de estudos experimentais que analisam toxicidade de substâncias ingeridas. Considera-se que a responsabilidade dos pesquisadores quanto às possíveis consequências aos participantes do estudo e os aspectos éticos envolvidos em delineamentos experimentais que avaliam toxicidade podem ser limitações para o desenvolvimento de pesquisas nessa área.

Em um relatório da Academia Americana de Pediatria, Trasande et al.^40^ (2018) discutem resultados de estudos sobre as consequências do consumo dos conservantes nitrito e nitrato à saúde das crianças. Os autores argumentam que algumas evidências apontam a ação desses conservantes como disruptores endócrinos, alterando o metabolismo tireoidiano e a interação com outras substâncias no organismo (aminas e amidas) formando compostos carcinogênicos, principalmente no cérebro e no trato gastrintestinal. Essa situação pode ser potencializada no organismo de lactentes e crianças pequenas, em função da imaturidade do organismo. Há, inclusive, evidências que destacam a relação entre consumo materno de nitritos e nitratos com o desenvolvimento de câncer cerebral em bebês.

Ocorrências de reações alérgicas em crianças pelo consumo de aditivos já foram publicadas cientificamente em relatos clínicos, principalmente associadas aos conservantes da classe dos benzoatos^112,113^, bem como aos corantes^114^. O consumo de corantes, especificamente, pode ativar a cascata inflamatória, resultando na indução da permeabilidade intestinal a grandes moléculas antigênicas. Além de reações alérgicas, a permeabilidade intestinal pode levar a doenças autoimunes e distúrbios neurocomportamentais^115^. Relato clínico sobre o tema aponta que não há dados de prevalência de alergia a aditivos alimentares em crianças, o que dificulta o diagnóstico. Entretanto, essa relação deve ser investigada clinicamente sempre que o paciente apresentar alergia a múltiplos alimentos e medicações^116^.

Há, ainda, estudos observacionais (populacionais e coorte) que encontraram possíveis correlações entre: consumo de edulcorantes artificiais e menarca precoce^117^; consumo de edulcorantes artificiais por mulheres grávidas e ganho de peso excessivo em bebês de até um ano^118^; e risco de sobrepeso em crianças de sete anos^119^; bem como consumo de glutamato monossódico, aspartame e nitritos como gatilhos para dores de cabeça em crianças^120^.

Pela já discutida questão ética de não ser adequado realizar estudos experimentais oferecendo aditivos potencialmente tóxicos a seres humanos, os estudos observacionais longitudinais se configuram as mais importantes fontes de levantamento de evidências sobre o tema. Entretanto, ressalta-se a impossibilidade de inferir relação de causalidade nesse desenho de estudo, bem como a dificuldade de separar os efeitos à saúde advindos dos aditivos dos demais componentes dos alimentos que os contêm.

Conforme exposto, a maior parte dos estudos de revisão encontrados sobre consumo de aditivos e saúde humana abordam os efeitos do consumo de edulcorantes e conservantes em adultos, enquanto em crianças apenas os efeitos dos corantes artificiais foram analisados. Os desfechos de saúde mais relacionados em crianças foram os transtornos comportamentais e imunológicos, embora em adultos estudos apontam outras possíveis consequências, como desenvolvimento de cânceres no trato gastrintestinal, desregulação metabólica, ganho de peso e efeito cardiometabólico. Entretanto, considerando que há centenas de aditivos permitidos para uso no mundo, uma parcela mínima dessas substâncias é estudada e testada em humanos, especialmente em crianças. Ademais, não foram encontrados estudos que avaliassem o impacto à saúde devido à ingestão regular e cumulativa de aditivos alimentares em humanos.

Quando se trata de crianças, o contexto de recomendações de consumo e avaliação de toxicidade dos aditivos é ainda mais complexo, pois um aspecto importante, o estágio inicial da vida, não é considerado no estabelecimento de recomendações. A IDA, parâmetro de consumo máximo para idealmente não haver efeito tóxico, é estabelecida por miligramas de aditivo por quilo de peso, mas não fica claro qual valor de quilo de peso é utilizado como referência para estabelecer esse parâmetro. Assim, questiona-se se a relação mg/kg de peso é aplicada pelos fabricantes de alimentos industrializados, considerando um peso médio infantil ou, como consequência, a toxicidade dos aditivos alimentares maior em crianças. Ao tratar-se dos aditivos sem IDA estabelecida, essa situação torna-se ainda mais preocupante. Nesses casos, sua adição aos alimentos deve seguir as boas práticas de fabricação, ou seja, os aditivos podem ser adicionados em quantidade *quantum satis,* que é a menor quantidade possível para alcançar o efeito tecnológico desejado, sem alterar a identidade e a genuinidade do alimento conforme padrões de identidade e qualidade determinados por regulamentos específicos^24,66^. Em tais casos, não é possível identificar qual quantidade de aditivo é adicionada ao alimento e se essa quantidade pode ser tóxica para as crianças, assim como não se sabe quais efeitos da combinação desses aditivos entre si e com os que a legislação prevê um limite máximo de uso.

## CONCLUSÕES

Trata-se de cenário no qual a literatura aponta risco à saúde das pessoas e, em especial, das crianças, cujo dever de proteção deve ser ainda maior, com absoluta prioridade. Contudo, o estabelecimento de limite de consumo de aditivos, ou IDA, é realizado considerando efeitos identificados em estudos toxicológicos realizados, majoritariamente, em modelos animais. Quando aplicado a crianças, o contexto de limites de consumo e a avaliação de toxicidade dos aditivos têm maior complexidade, pois um aspecto importante, o estágio inicial da vida, não é considerado no estabelecimento de limites de segurança. Sabe-se que a toxicidade dos aditivos alimentares é maior em crianças, em virtude de a quantidade ingerida por quilo de peso ser maior. Ademais, os órgãos e sistemas estão ainda em formação nesse estágio da vida, expondo as crianças a potenciais riscos maiores à saúde que podem advir do consumo de aditivos. Além disso, o nível de exposição ao longo da vida pode ser maior em crianças atualmente, já que iniciaram o consumo de alimentos industrializados e aditivos alimentares ainda nos primeiros anos de vida^31,32^.

Nesse sentido, considera-se evidente a limitação existente no método científico para realizar estudos de toxicidade de substância potencialmente tóxicas em humanos, especialmente em crianças. Adicionalmente, ressalta-se haver limitações metodológicas para a avaliação do consumo de aditivos por crianças, primeiramente, porque os métodos de avaliação do consumo alimentar são diversos e nem sempre comparáveis, somando-se ao fato de que a quantificação dos aditivos nos alimentos é realizada de diferentes maneiras, sendo a análise laboratorial considerada o padrão-ouro. Contudo, muitos estudos estimam a quantidade de aditivos nos alimentos, por meio do limite máximo permitido para cada substância, ocasionando divergências metodológicas que dificultam a comparação dos resultados dos estudos, bem como a análise da qualidade metodológica. Assim, entende-se que esse contexto contribui para a fragilidade das evidências existentes, bem como para a escassez de discussões acerca do tema.

Desse modo, com base no princípio da precaução, cabe ao Estado promover medidas que visem proteger a saúde da população (incluindo o risco, nos termos do artigo 196 da Constituição e 9^o^ do Código de Defesa do Consumidor), o que resulta no dever de promover o debate público sobre o tema e políticas públicas que permitam o acesso a informações sobre a quantidade de aditivo utilizada nos alimentos, de modo que as pessoas possam fazer as suas escolhas informadas e conscientes.

Observa-se que os estudos sobre consumo de aditivos, bem como aqueles que avaliaram consequências à saúde, concentram suas análises em três classes funcionais: corantes, edulcorantes e conservantes. Entretanto, questiona-se a representatividade dos aditivos estudados frente ao total de aditivos permitidos para uso. No Brasil, há 23 classes funcionais regulamentadas e centenas de normas da Anvisa que estabelecem quais aditivos e em qual quantidade podem ser utilizados nos alimentos^e^. Esse contexto inviabiliza a análise precisa de quantos aditivos são permitidos para uso no país, para que seja possível verificar o cenário de discussões científicas acerca do tema. Adicionalmente, a análise da notificação de aditivos em rótulos de alimentos industrializados é escassa no Brasil e no mundo. Por meio desses dados, seria possível avaliar quais aditivos são utilizados com maior frequência nos alimentos industrializados e, assim, relacionar dados de frequência de uso, consumo e consequências à saúde.

Por fim, os aditivos, como os corantes e edulcorantes, estão presentes não apenas em alimentos, mas também em medicamentos e produtos de higiene bucal, podendo ser ingeridos por meio de diversas fontes. Assim, pondera-se a relevância de um debate técnico-científico ampliado quanto ao estabelecimento de parâmetros mais rígidos de consumo e toxicidade de aditivos específicos para crianças, considerando as diversas fontes de exposição a essas substâncias.

## Footnotes

a Parte da denominada classificação NOVA dos alimentos. Os ultraprocessados são alimentos usualmente fabricados a partir de múltiplos ingredientes. Esses alimentos podem conter ingredientes extraídos de outros alimentos (caseína, glúten etc.) ou derivados de outros alimentos (maltodextrina, açúcar invertido, óleos hidrogenados etc.). Ademais, aditivos alimentares como corantes, edulcorantes, estabilizantes, realçadores de sabor, entre outros, são utilizados de maneira intensiva.

b Sigla do inglês *buthylated hydroxyanisole*

c Sigla do inglês *butylated hydroxytoluene*

d Sigla do inglês *tertiary butylhydroquinone*

e A Anvisa está em processo de compilação das normas que regulamentam o uso de aditivos em alimentos industrializados no Brasil.
